# Kolmogorov-Smirnov-type test for dependently double-truncated durations: A copula approach

**DOI:** 10.1007/s10985-026-09722-0

**Published:** 2026-07-18

**Authors:** Anne-Marie Toparkus, Rafael Weißbach

**Affiliations:** Chair of Statistics and Econometrics, Faculty for Economic and Social Sciences, University of Rostock, 18051 Rostock, Germany

**Keywords:** Double truncation, Dependent truncation, Goodness-of-fit, Copula, Kolmogorov-Smirnov, 62N03, 62N05, 62D10

## Abstract

Given a double-truncated sample of lifespans, we test the hypothesis of a parametric distribution family for the lifespan. Demography typically certifies the life expectancy to be nonstationary in time. We model the resulting dependence between a lifespan and the birthday of an individual with a copula. Our main example is the Farlie-Gumbel-Morgenstern copula. The asymptotic null distribution of the test is based on Donsker-class arguments and the functional delta method for empirical processes. One assumption for the test is consistency in the estimation of the parameter for the model under the null hypothesis. Requirements for consistency are fulfilled by our set of assumptions. The test is carried out in two stages, the computation of the test statistics and the simulation of the critical value. The statistic can be reached after finitely many computation steps, whereas the critical value can only be an approximation. With the exponential distribution as an example for the lifespan distribution, and for the application to 55,000 German double-truncated enterprise lifespans, the constructed Kolmogorov-Smirnov test rejects clearly an age-homogeneous closure hazard.

## Introduction

In the frequent situation of right censoring, a lifespan is not precisely observed if life ends after the study. Whilst in the case of censoring, at least some information about the individual is known, in the case of truncation, no information at all is available, not even about its existence. As a consequence, the sample size will only be latent and to be distinguished from the number of observations. The reason for the absence of information is similar for censoring and truncation, namely that a life ends outside the study period, being typically an interval with open ends at the top (“left truncation”) or the bottom (“right truncation”) (see Lawless 2003). Left truncation occurs frequently in addition to right censoring in medical or social science applications. The literature on their joint analysis is correspondingly extensive (see e.g. Klein and Moeschberger 2003; Turnbull 1976; Gross and Lai 1996). If right truncation occurs jointly with left truncation, this is referred to as double truncation. Such cases are found, for example, in epidemiological studies or in economic applications, as in the present work. The statistical treatment of double truncation also has a long history (including Efron and Petrosian 1999; Shen 2010; Moreira and de Uña-Álvarez 2010).

Nonparametric calculations for truncated lifespans are not able to estimate life expectancy, or influences on it, both being popular goals in applied statistics. Therefore, Weißbach and Wied (2022) adopt a parametric approach. Their model for the survival time *X* of an enterprise is the exponential distribution, whereas the founding date, coded as the age at the start of the study *T*, is assumed to be uniformly distributed. The age at the study start decides about truncation (see Weißbach and Wied 2022, Figure1). In this and many other studies, it is assumed for simplicity that the lifespan of interest and the truncation age are stochastically independent. Particularly in the context of human or business demography, independence contradicts the established finding that lifespans increase in time, i.e. depend on the date of birth.

Various non- and semi-parametric approaches for dependent truncation already exist (Akritas and LaValley 2005; Efron and Petrosian 1994; Chiou et al. 2019). The latter two works are related to the idea of circumventing a sample selection bias with the help of covariates, as in the popular propensity score method. Our approach does not rely on a covariate. We describe dependence with a copula (following Chaieb and Rivest 2006; Emura and Wang 2012; Moreira et al. 2021) and are comparable to Emura and Pan (2020) who use the copula approach to perform likelihood inference in the case of dependent left truncation. (For the potential range of applications see Ma and Zhang (2022), who analyse ocean data.) We will make some use of the previous study (Toparkus and Weißbach 2025), that parametrically extends Weißbach and Wied (2022) to dependent truncation with a copula introduced in Gumbel (1960), however our copula here will be different. We will use the Farlie-Gumbel-Morgenstern copula, which allows positive as well as negative dependence, and even independence. Its analytical tractibility permits explicit derivations of the covariance structure. The copula will however not be suited for strong correlation or tail dependence, as can be found e.g. between macroeoconomic measurements. The approach is most suitable for situations involving weak to moderate dependence.

We aim in this paper at testing the parametric assumption about the distribution, and build on the nonparametric and asymptotic Kolmogorov-Smirnov test (starting with Kolmogorov 1933). Emura et al. (2015) followed the same fundamental idea for independent left-truncation, however for a non-composite hypothesis, i.e. not taking account of the parameter estimation under the hypothesis. Our hypothesis will be composite and take account of estimating unknown parameters as well as the latent sample size. For truncation with its outcomes *X* and *T*, the test is two-dimensional and the calculation of the test statistic itself proves to be computationally intensive. And unlike in the one-dimensional case, the limit distribution of the test statistic in the multivariate case depends on the underlying distribution, or at least on the copula it contains. For the two-dimensional case, the Kolmogorov distribution can still be used to calculate the limiting distribution, at least given the independence of the individual random variables (Naaman 2021). An alternative approach to our, also for truncation dependence, is Kojadinovic and Yan (2011) who use the so-called multiplier method. In this method, random weights, known as multipliers, are generated to simulate a weighted version of the empirical process. Emura and Konno (2012) also make use of this method to perform a goodness-of-fit test for dependent left truncation. In their work, they already emphasize that conventional bootstrap procedures are computationally intensive and therefore inefficient. Nevertheless, the multiplier method is based on the given data structure and may vary depending on the sample. Even though also our critical value must also be simulated, its simulation only depends on the explicitly derived asymptotic distribution and is very simple.

In Sect. 2, we define the bivariate distribution model of lifespan and truncation age in the population, formalize the sampling design, derive the observation probability and finally describe the test statistic. Section 3 applies an algorithm of Justel et al. (1997) in order to reduce the computation of the statistic to finitely many operations. Section 4 applies Donsker arguments and the functional delta method to find the asymptotic distribution of the test statistic under both truncation independence and dependence, and derives an algorithm for the critical value. Section 5 derives, for the example of an exponentially distributed lifespan, the score function for the estimating equation and the covariance function of the asymptotic test distribution. The simulation study in Sect. 6 finds actual size and power for medium sample sizes to be appropriate. Section 7 applies the results to the dataset in Weißbach and Wied (2022) and rejects the assumption of an exponential distribution as model for enterprise lifespans in Germany, as long as specification of dependence and foundation distribution hold true.

## Population model, sampling design and test statistic

The population of interest are *G* birth cohorts (*0< G < ∞ *). The lifespan *X* with density $f_{\theta }^X$ is of interest and the date of birth will also prove to be relevant. Instead of measuring the latter in calender time, its is counted backwards from the end of the birth period, *T ∈ [0,G]*, with density $f^T$ and cumulative distribution $F^T$. The domain of outcomes is $S:=\mathbb {R}^+_0\times [0,G]$, which we equip with the Borel sigma-field $\mathcal {A}$. We assume dependence between *X* and *T* and describe it with the copula $C_{\vartheta }$. The bivariate distribution of $(X,T)^\text {T}$ now depends on the parameter $\boldsymbol{\theta }:=(\theta , \vartheta )^\text {T} \in \Theta$, of dimension two. The joint distribution function is, according to Sklar’s theorem (Nelsen 2006, Theorem 2.3.3), $F_{\boldsymbol{\theta }}(x,t):=C_{\vartheta }(F^X_{\theta }(x),F^T(t))$, with density $f_{\boldsymbol{\theta }}$ and probability measure $\mathbb {P}_{\boldsymbol{\theta }}$. We assume the existence of a latent sample, a simple random sample consisting of $n\in \mathbb {N}$ random variables $\Omega \rightarrow S$ with $\mathbb {P}_{\boldsymbol{\theta }_0}$, i.e. $(X_i,T_i)^\text {T}, \, i=1, \ldots , n$, with the unknown true parameter $\boldsymbol{\theta }_0 \in \Theta$.

The study starts immediately after the birth period and includes a sample individual if its has not died before the study (left truncation). Let the study continues for *s>0* time units, and also exclude any individual that dies afterwards (right truncation) (for more motivation see Weißbach and Wied 2022). Note that *T* now has the interpretation as age-at-study-start. Formally, $(X_i,T_i)^\text {T}$ is observed if it is in the parallelogram

1 $$\begin{aligned} D:=\{(x,t)^\text {T}|0<t\le x\le t+s,t\le G\}\subset S. \end{aligned}$$

An observation may be denoted by $(X_j^{\text {obs}},T_j^{\text {obs}})^\text {T}$ with ${j=1,\ldots ,M_n\le n}$, but consider that these are not measurable mappings $\Omega \rightarrow \mathcal {A}$. Furthermore, note also that $M_n=\sum _{i=1}^n 1\!\!1_{\{(X_i,T_i)^\text {T} \in D\}}$ is random. The probability that the *i*th sample individual is observed can be calculated through

$$\begin{aligned} \alpha _{\boldsymbol{\theta }}:=\mathbb {P}_{\boldsymbol{\theta }}(T_i\le X_i\le T_i +s)=\int _0^G\int _t^{t+s} f_{\boldsymbol{\theta }}(x,t) \,\textrm{d}x \,\textrm{d}t. \end{aligned}$$

For a left-truncated design, Emura and Pan (2020, Theorem 1) provides a method for reducing the integral to one dimension. This saves computational time, and Toparkus and Weißbach (2025, Equation 2) derive for the dependently double-truncated design

2 $$\begin{aligned} \alpha _{\boldsymbol{\theta }}=\int _0^1 \left[ c_u\{F^T((F^X_{\theta })^{-1}(u))\}-c_u\{F^T((F^X_{\theta })^{-1}(u))-F^T(s)\} \right] \,\textrm{d}u, \end{aligned}$$

where $c_u(v):=\frac{\partial C_{\vartheta }(u,v)}{\partial u}$. We assume that

1. $$0< \alpha _{\boldsymbol{\theta }} <1$$, for all $$\boldsymbol{\theta }\in \Theta $$.

A Kolmogorov-Smirnov (KS) test makes use of the distance between a nonparametric and the parametric estimate of a distribution. A supremum is taken, so that for a required asymptotic distribution of the test statistic, the distance function needs not only to converge pointwise, but rather uniformly. Instead of thinking of the supremum to be taken, in the two dimensions *x* and *t*, or over rectangles *[0, x]× [0,t]*, we think of a supremum taken over functions $1\!\!1_{[0, x]\times [0,t]}$. Other than for a simple hypothesis, of a parametric distribution with known parameter, we formulate the composite hypothesis, with estimated parameter. This will require the functional delta method and the following assumptions will (i) enable a simple calculation of the Fréchet derivative, (ii) can easily be verified, and (iii) do not rule out any model which we have encountered so far. Recall that a partially differentiable function with continuous derivatives is totally differentiable. Therefore, we write, in short, “continuously differentiable”. (As usual, $\dot{g}_{\theta }$ and $\dot{g}_{\vartheta }$ denote the partial derivatives $\frac{\partial }{\partial \theta } g_{\boldsymbol{\theta }}$ and $\frac{\partial }{\partial \vartheta } g_{\boldsymbol{\theta }}$, respectively, and $\dot{g}_{\boldsymbol{\theta }}:=(\dot{g}_{\theta },\dot{g}_{\vartheta })^{\text {T}}$ is the gradient, for whatever function $g_{\boldsymbol{\theta }}$.)

(A2) The function $(x,t) \mapsto f_{\boldsymbol{\theta }}(x,t)$ is integrable for each $\boldsymbol{\theta }\in \Theta$; the map $\boldsymbol{\theta }\mapsto f_{\boldsymbol{\theta }}(x,t)$ is continuously differentiable on *Θ * for each *(x,t) ∈ S* with the derivatives $\dot{f}_{\boldsymbol{\theta }}:=(\dot{f}_{\theta },\dot{f}_{\vartheta })^\text {T}$.

Let $\hat{\boldsymbol{\theta }}_n$ be a reasonable estimator for $\boldsymbol{\theta }_0$. It is not required that $\hat{\boldsymbol{\theta }}_n$ be a maximum likelihood estimator; however consistency and asymptotic linearity are essential.

(A3) It is $\hat{\boldsymbol{\theta }}_n \overset{P}{\longrightarrow }\boldsymbol{\theta }_0$ as $n \rightarrow \infty$.
(A4) With some $\phi _{\boldsymbol{\theta }_0}=(\phi _{\boldsymbol{\theta }_0,1},\phi _{\boldsymbol{\theta }_0,2})^{\text {T}}$, the estimating sequence is asymptotically linear: $\sqrt{n}(\hat{\boldsymbol{\theta }}_n-\boldsymbol{\theta }_0)=\frac{1}{\sqrt{n}} \sum _{i=1}^n \phi _{\boldsymbol{\theta }_0}(X_i,T_i)+o_{\mathbb {P}_{\boldsymbol{\theta }_0}}(1)$
(A5) The map $\boldsymbol{\theta }\mapsto \phi _{\boldsymbol{\theta }}(x,t)$ is continuously differentiable and fulfills $\sup _{(x,t) \in S} \Vert \phi _{\boldsymbol{\theta }_0}(x,t)\Vert < \infty$, $\mathbb {E}_{\boldsymbol{\theta }_0}[\phi _{\boldsymbol{\theta }_0}(X_i,T_i)1\!\!1_{ D}(X_i,T_i)] ={\textbf {0}}$, $\mathbb {E}_{\boldsymbol{\theta }_0}[\phi _{\boldsymbol{\theta }_0}(X_i,T_i)]={\textbf {0}}$ and $\mathbb {E}_{\boldsymbol{\theta }_0}[\Vert \phi _{\boldsymbol{\theta }_0}(X_i,T_i)\Vert ^2]<\infty$.

Approaches to derive the function $\phi _{\boldsymbol{\theta }}$ and prove Assumptions (A3) and (A4) for independent and dependent double truncation can be found in Weißbach and Wied (2022) and Toparkus and Weißbach (2025), respectively.

The statistic of the KS test in order to test

3 $$\begin{aligned} \begin{aligned}&H_0: \, \text {The distribution of } (X_i,T_i)^{\text {T}} \text { derives from the parametric family}\\&\, \qquad \{F_{\boldsymbol{\theta }}=C_{\vartheta }(F_{\theta }^X,F^T) \, |\, \boldsymbol{\theta }\in \Theta \}.\\&H_1: \, \text {The distribution of } (X_i,T_i)^{\text {T}} \text { does not derive from the parametric}\\&\, \qquad \text {family }\{F_{\boldsymbol{\theta }}=C_{\vartheta }(F_{\theta }^X,F^T)\,|\, \boldsymbol{\theta }\in \Theta \}. \end{aligned} \end{aligned}$$

has two dimensions. The distribution of an observation $(X_j^{\text {obs}}, T_j^{\text {obs}})^\text {T}$ is

4 $$\begin{aligned} F_{\boldsymbol{\theta }}^{\text {obs}}(x,t):=\mathbb {P}_{\boldsymbol{\theta }}((X_i,T_i)^\text {T}\in [0,x]\times [0,t] \cap D)/\alpha _{\boldsymbol{\theta }}. \end{aligned}$$

Note that $(X_j^{\text {obs}}, T_j^{\text {obs}})^\text {T}$ and $(X_{j'}^{\text {obs}}, T_{j'}^{\text {obs}})^\text {T}$ are not independent vectors for *j≠ j'*. The unobservable empirical distribution $$\mathbb {P}_n(A):=n^{-1}\sum _{i=1}^n \epsilon _{\left( {\begin{array}{c}X_i\\ T_i\end{array}}\right) }(A)$$ for *A:=[0,x] × [0,t]* remains unobservable even if changed to

$$ \mathbb {P}_{n,D}(A):=n^{-1}\sum _{i=1}^n \epsilon _{\left( {\begin{array}{c}X_i\\ T_i\end{array}}\right) }(A \cap D), $$

which estimates the distribution only on the observable support. The comparison with its parametric analogue $\alpha _{\boldsymbol{\theta }_0} F_{\boldsymbol{\theta }_0}^{\text {obs}}(x,t)$ results in a KS test statistic, for known *n* and a known true parameter $\boldsymbol{\theta }_0$, as

5 $$\begin{aligned} \sup _{(x,t)^{\text {T}} \in S} \sqrt{n} \, \big \vert \mathbb {P}_{n,D}([0,x]\times [0,t])- \alpha _{\boldsymbol{\theta }_0}F_{\boldsymbol{\theta }_0}^{\text {obs}}(x,t) \big \vert . \end{aligned}$$

We also take into account that neither $\boldsymbol{\theta }_0$ nor *n* are observed in practical applications. (In a parametric analysis, at least *n* can be profiled out and does not need to be estimated (see Weißbach and Wied 2022).) Here we use $\hat{n}:=M_n/\alpha _{\hat{\boldsymbol{\theta }}_n}$, the maximum likelihood estimator. Inserting the estimators leads to the composite KS statistic:

6 $$\begin{aligned} \frac{\sqrt{M_n}}{\sqrt{\alpha _{\hat{\boldsymbol{\theta }}_n}}} \sup _{(x,t)^{\text {T}} \in S} \left| \mathbb {P}_{\hat{n},D}\big ([0,x]\times [0,t]\big )- \alpha _{\hat{\boldsymbol{\theta }}_n} F_{\hat{\boldsymbol{\theta }}_n}^{\text {obs}}(x,t)\right| \end{aligned}$$

with $$\mathbb {P}_{\hat{n},D}:= \frac{\alpha _{\hat{\boldsymbol{\theta }}_n}}{M_n} \sum _{i=1}^n \epsilon _{\left( {\begin{array}{c}X_i\\ T_i\end{array}}\right) }(A \cap D)= \frac{\alpha _{\hat{\boldsymbol{\theta }}_n}}{M_n} \sum _{j=1}^{M_n} \epsilon _{(X_j^{\text {obs}},T_j^{\text {obs}})^{\text {T}}}(A)$$.

## Computation of the test statistic

Recall (4), using the observations, the test statistic (6) can then be rewritten as

$$\begin{aligned}&\frac{\sqrt{M_n}}{\sqrt{\alpha _{\hat{\boldsymbol{\theta }}_n}}} \sup _{(x,t)^{\text {T}} \in S} \left| \mathbb {P}_{\hat{n},D}\big ([0,x]\times [0,t]\big )-\mathbb {P}_{\hat{\theta }_n}\big ((X_i,T_i)^\text {T} \in [0,x]\times [0,t]\cap D\big )\right| \\&\quad = \sqrt{\alpha _{\hat{\boldsymbol{\theta }}_n}}\sqrt{M_n} \sup _{(x,t)^{\text {T}} \in D} \left| \frac{1}{M_n} \sum _{j=1}^{M_n} \epsilon _{{{X_j^{\text {obs}}} \atopwithdelims (){T_j^{\text {obs}}}}}([0,x]\times [0,t]) - F^{\text {obs}}_{\hat{\boldsymbol{\theta }}_n}(x,t) \right| . \end{aligned}$$

If the distribution was of dimension one, then, due to monotonicity of the CDF, the univariate Kolmogorov-Smirnov test statistic could be obtained by computing the distance at the finitely many discontinuity points of the empirical distribution. This would include the right-side limits at these points (see e.g. Hollander et al. 2014). The monotonicity argument also holds true in the bivariate case, with the two dimensions $x^{\text {obs}}$ and $t^{\text {obs}}$. However, the set of discontinuities is not finite, but, again by virtue of monotonicity arguments, the calculation of the KS statistic requires only a finite number of points when computing (6). Justel et al. (1997) present details for two independent uniform distributions, with the unit square as support. We adjust their arguments to the parallelogram *D* as support.

Let $(x_1^{\text {obs}},t_1^{\text {obs}})^{\text {T}}, \ldots , (x_{m_n}^{\text {obs}},t_{m_n}^{\text {obs}})^{\text {T}}$ be the realization of a truncated sample and let $F^{\text {obs}}_{m_n}$ denote the realized empirical distribution function. Define left-side and right-side differences

$$\begin{aligned} D_{m_n}^+(x,t):= F^{\text {obs}}_{m_n} (x, t)-F^{\text {obs}}_{\hat{\boldsymbol{\theta }}_n}(x,t) \quad \text {and} \quad D_{m_n}^-(x,t):= F^{\text {obs}}_{\hat{\boldsymbol{\theta }}_n}(x,t)- F^{\text {obs}}_{m_n}(x, t). \end{aligned}$$

**Fig. 1:**
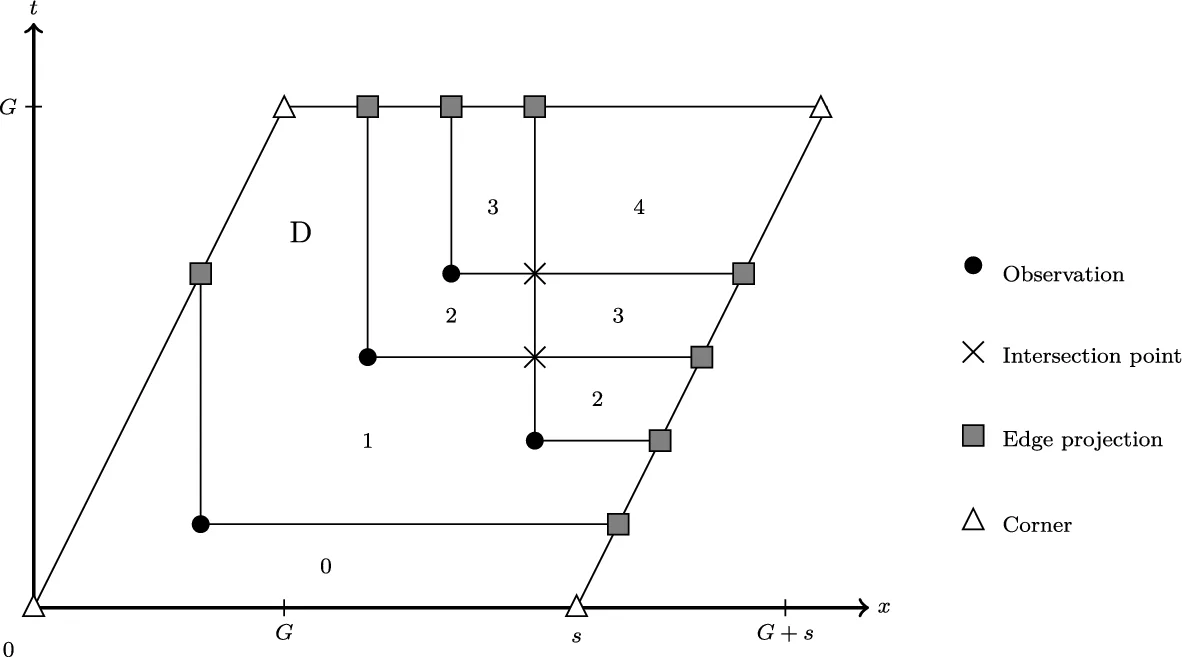
Two-dimensional empirical distribution function $F^{\text {obs}}_{m_n}$ for $m_n=4$ (multiplied by 4) and observable support under double truncation parallelogram $D=\{(x,t)^\text {T}|t\le x\le t+s\}$ (see (1))

Then, the KS statistic (6) needs the maximum of $\delta _{m_n}^+:= \sup _{(x,t)^{\text {T}} \in D} D_{m_n}^+(x,t)$ and $\delta _{m_n}^-:= \sup _{(x,t)^{\text {T}} \in D} D_{m_n}^-(x, t)$. Similar to Justel et al. (1997), we can obtain the left-side difference $\delta _{m_n}^{+}$ as the maximum of $D_{m_n}^+(x,t)$ evaluated at (0, 0), the observation points (see *∙ * in Fig. 1) and the intersections originating from discordant points,

$$\begin{aligned} I:=\{(x_j^{\text {obs}}, t_{j'}^{\text {obs}}) \vert \,x_{j'}^{\text {obs}} < x_j^{\text {obs}}, \; t_{j'}^{\text {obs}} > t_j^{\text {obs}}; \, j, j'=1, \ldots , m_n\} \end{aligned}$$

(see *× * in Fig. 1). For $\delta _{m_n}^-$ we need to evaluate the difference $F^{\text {obs}}_{\hat{\boldsymbol{\theta }}_n}(x,t)- F^{\text {obs}}_{m_n}(x^-, t^-)$ with the definition $F^{\text {obs}}_{m_n}(x^-, t^-):= \lim _{\delta \searrow 0}F^{X^{\text {obs}}, T^{\text {obs}}}_{m_n}(x- \delta , t-\delta )$, at *(G+s,G)*, the intersection points and the projection points of the observed points onto the upper and right edge of *D*,

$$\begin{aligned} P:=\{(x_j^{\text {obs}}, \min (x_j^{\text {obs}}, G)) \vert j=1, \ldots , m_n\} \cup \{(t_j^{\text {obs}}+s, t^{\text {obs}}_j) \vert j=1, \ldots , m_n\} \end{aligned}$$

(see ◾; in Fig. 1), and three of the corners, namely $(G,0)^{\text {T}}, (s,G)^{\text {T}},$ and $(G+s,G)^{\text {T}}$ (see *△ * in Fig. 1).

Depending on the sample, $3m_n$ to $$3m_n+\left( {\begin{array}{c}m_n\\ 2\end{array}}\right) $$ evaluations are required. Fortunately, the evaluations for $D_{m_n}^-$ can be traced back to $D_{m_n}^+$. For the set of intersection points *I*, it is for instance:

$$\begin{aligned}&\max _{(x,t)^{\text {T}} \in I}(F^{X^{\text {obs}}, T^{\text {obs}}}_{\hat{\boldsymbol{\theta }}_n}(x,t)-F^{X^{\text {obs}}, T^{\text {obs}}}_{m_n}(x^-, t^-)) \\&=-\min _{(x,t)^{\text {T}} \in I}(F^{X^{\text {obs}}, T^{\text {obs}}}_{m_n}(x^-, t^-)-F^{X^{\text {obs}}, T^{\text {obs}}}_{\hat{\boldsymbol{\theta }}_n}(x, t)) \\&=-\min _{(x,t)^{\text {T}} \in I}\left[ \frac{1}{m_n}\sum _{j=1}^{m_n}\epsilon _{\left( {\begin{array}{c}x_j^{\text {obs}}\\ t_j^{\text {obs}}\end{array}}\right) }\big ([0,x)\times [0,t) \big ) -F^{X^{\text {obs}}, T^{\text {obs}}}_{\hat{\boldsymbol{\theta }}_n}(x, t)\right] \\&=\frac{2}{m_n}-\min _{(x,t)^{\text {T}} \in I}\left[ \frac{1}{m_n}\sum _{j=1}^{m_n}\epsilon _{\left( {\begin{array}{c}x_j^{\text {obs}}\\ t_j^{\text {obs}}\end{array}}\right) }\big ([0,x]\times [0,t] \big ) -F^{X^{\text {obs}}, T^{\text {obs}}}_{\hat{\boldsymbol{\theta }}_n}(x, t)\right] \\&=\frac{2}{m_n}-\min _{(x,t)^{\text {T}} \in I}\left[ F^{X^{\text {obs}}, T^{\text {obs}}}_{m_n}(x, t)-F^{X^{\text {obs}}, T^{\text {obs}}}_{\hat{\boldsymbol{\theta }}_n}(x,t)\right] . \end{aligned}$$

Therefore, the test statistic can be obtained in eight steps. 

**Algorithm 1:**
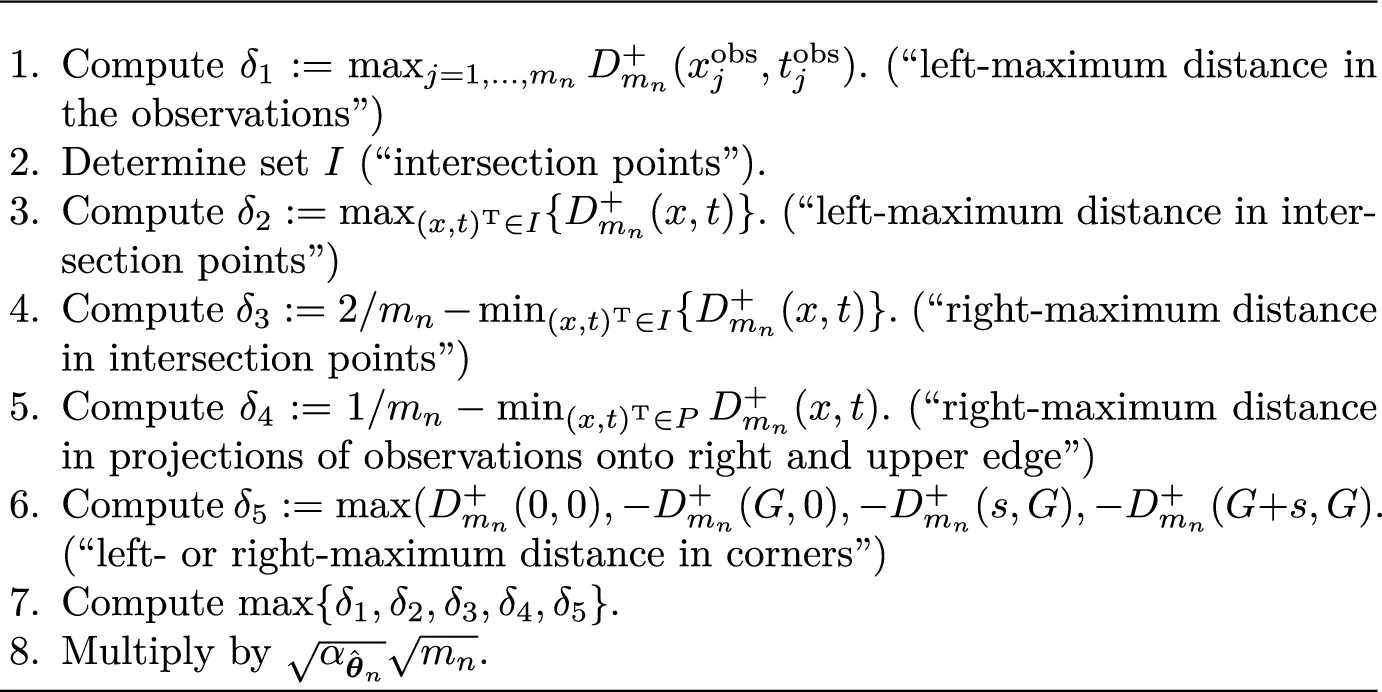
Computation of KS test statistic (6)

As a strategy to make the computation feasible, the computation of intersection points can be restricted to discordant pairs only, i.e., pairs satisfying $X_i<X_j$ and $T_i>T_j$, which substantially reduces the number of candidate points compared to the worst-case quadratic complexity. Furthermore, duplicate intersection points can be removed prior to evaluation, ensuring that each point is processed only once. In addition, boundary evaluations are performed only at unique values of the observed data, avoiding redundant computations. The implementation can rely on vectorized operations and efficient data structures, which can further improve computational performance in large samples.

## Distribution of the test statistic under **H**_0_

After the computation of our test statistic, we now derive the critical value, i.e. the quantile of (6) under $H_0$ of (3). Section 4.1 contains some preparations. It can be shown that these are sufficient for the non-composite case where the latent sample size and the parameter are known. Section 4.2 still considers *n* to be known but $\boldsymbol{\theta }_0$ to be estimated and Sect. 4.3 finally treats both simultaneously as unknown.

### Limit theorems for truncated data

Consider set *B ⊂ S* as a “point”; then $\mathbb {P}_{n,D}(B)$ and $\sqrt{n} \mathbb {P}_{n,D}(B)$ converge pointwise by the LLN and the CLT. An important set will be *[0,x]× [0,t]*. The test statistic (6) includes a supremum, so that convergence needs to be uniform, or in other words, functional. (Note again that a corresponding supremum will finally not be one of points in a set, neither one of sets within some algebra, but a supremum of functions $1\!\!1_{[0, x]\times [0,t] \cap D}$.) We switch to the functional-valued notation (see van der Vaart (1998, Sect. 19.2) or van der Vaart and Wellner (1996, Sect. 2.1)).

The empirical distribution $\mathbb {P}_n$ has been defined in Sect. 2, and for a given class $\mathcal {G}$ of Borel-measurable functions $g: S \rightarrow \mathbb {R}$, denote $\mathbb {P}_n (g)$ the expectation of *g* under $\mathbb {P}_n$, i.e.

$$\begin{aligned} g \mapsto \mathbb {P}_n (g):=\frac{1}{n} \sum _{i=1}^n g(X_i,T_i). \end{aligned}$$

Furthermore, define $\mathbb {E}_{\boldsymbol{\theta }}(g):=\mathbb {E}_{\boldsymbol{\theta }}(g(X_i,T_i))=\int _{\Omega } g(X_i,T_i) \,\textrm{d}\mathbb {P}_{\boldsymbol{\theta }}$. The centered and scaled version of the mapping $g \mapsto \mathbb {P}_n (g)$ is the (functional-valued) *empirical process* evaluated in *g*, that is:

$$\begin{aligned} g \mapsto \mathbb {G}_n( g):=\sqrt{n}(\mathbb {P}_n-\mathbb {E}_{\boldsymbol{\theta }_0})(g)=\frac{1}{\sqrt{n}} \sum _{i=1}^n (g(X_i,T_i)-\mathbb {E}_{\boldsymbol{\theta }_0}(g)) \end{aligned}$$

(We will also use $g \mapsto \mathbb {G}_n( g)$ for two-dimensional functions $g=(g_1,g_2)^\text {T}$, for which $\mathbb {P}_n(g)=(\mathbb {P}_n(g_1),\mathbb {P}_n(g_2))^\text {T}$ will be calculated component-wise, as is common for expectations.) For the functional-valued Brownian bridge, two classes of functions will become important (see e.g. van der Vaart and Wellner 1996, Sect. 2.1). A class $\mathcal {G}$ of measurable functions $g: S \rightarrow \mathbb {R}$ is called *Glivenko-Cantelli* if $n^{-1/2}\Vert \mathbb {G}_n\Vert _{\mathcal {G}}:=n^{-1/2} \sup _{g\in \mathcal {G}} \vert \mathbb {G}_n( g) \vert \longrightarrow 0$, where the convergence is outer almost surely as $n \rightarrow \infty$. Further details about outer integrals can be found, for example, in van der Vaart and Wellner (1996, Sect. 1.2). A class $\mathcal {G}$ of measurable functions $g: S\rightarrow \mathbb {R}$ is called *Donsker*, if the sequence of processes $\{\mathbb {G}_n(g): \, g \in \mathcal {G}\}$ converges in distribution to a tight limit process $\mathbb {G}$ in the space $\ell ^{\infty }(\mathcal {G})$. Then, the limit process is Gaussian with zero mean and covariance function

7 $$\begin{aligned} \text {Cov}[\mathbb {G}(g), \mathbb {G}(h)]=\mathbb {E}_{\boldsymbol{\theta }_0}(gh)-\mathbb {E}_{\boldsymbol{\theta }_0}(g)\mathbb {E}_{\boldsymbol{\theta }_0}(h) \end{aligned}$$

and therefore especially a $\mathbb {P}_{\boldsymbol{\theta }_0}$-Brownian bridge with $\mathbb {B}_{\mathbb {P}_{\boldsymbol{\theta }_0}}(g):=\mathbb {B}(\mathbb {E}_{\boldsymbol{\theta }_0}(g))$, with $\mathbb {B}$ as real-valued Brownian bridge on [0, 1].

According to van der Vaart (1998, Ex. 19.6) or van der Vaart and Wellner (1996, Ex. 2.5.4) the class of functions $\mathcal {G}:=\{g_{x,t}=1\!\!1_{[0, x]\times [0,t] }, \, (x,t) \in S\}$ is Glivenko-Cantelli and Donsker. In view of our truncated process, we need to multiply each function with $1\!\!1_D$. A certain kind of Lipschitz condition ensures that the desired properties will extend.

#### Corollary 1

The class of functions $\mathcal {G}_D:=\{g_{x,t,D}=1\!\!1_{[0, x]\times [0,t] }1\!\!1_D, \, (x,t) \in S\}$ is Glivenko-Cantelli and Donsker

#### Proof

As stated above, the class $\mathcal {G}$ is Donsker with $\Vert \mathbb {E}_{\boldsymbol{\theta }_0}\Vert _{\mathcal {G}}\le 1<\infty$. Furthermore, the function $(x,t) \mapsto 1\!\!1_D(x,t)$ is measurable and uniformly bounded. Although the product of functions from $\mathcal {G}$ and $1\!\!1_D$ is not a Lipschitz function, we have

$$\begin{aligned} \vert g_{x_1,t_1,D}(x,t) 1\!\!1_D(x,t)&- g_{x_2,t_2,D}(x,t) 1\!\!1_D(x,t)\vert \\&\le \Vert 1\!\!1_D \Vert _{\infty }\, \vert g_{x_1,t_1,D}(x,t) - g_{x_2,t_2,D}(x,t) \vert \end{aligned}$$

for all $(x,t), (x_1,t_1), (x_2,t_2) \in S$. Hence, van der Vaart and Wellner (1996, Condition (2.10.5)) is satisfied and Theorem 2.10.6 applies therein. Hence, the class $\mathcal {G}_D$ is Donsker. It is also Glivenko-Cantelli, given that every Donsker class is a Glivenko-Cantelli class (van der Vaart and Wellner, 1996, Sect. 2.1). *□ *

In terms of the truncated empirical distribution, this means that

$$\begin{aligned} \sup _{(x,t) \in S} \big \vert \mathbb {P}_{n,D}\big ([0,x]\times [0,t]\big )-\mathbb {P}_{\boldsymbol{\theta }_0}\big ((X_i,T_i)^\text {T} \in [0,x]\times [0,t]\cap D\big ) \big \vert \overset{a.s.}{\longrightarrow }0 \end{aligned}$$

and that the sequence

$$\begin{aligned} \big \{\sqrt{n}\big (\mathbb {P}_{n,D}\big ([0,x]\times [0,t]\big )-\mathbb {P}_{\boldsymbol{\theta }_0}\big ((X_i,T_i)^\text {T} \in [0,x]\times [0,t]\cap D\big )\big ): (x,t) \in S\big \} \end{aligned}$$

converges in distribution to a $\mathbb {P}_{\boldsymbol{\theta }_0}$-Brownian bridge $\mathbb {B}_{\mathbb {P}_{\boldsymbol{\theta }_0}}(g_{x,t,D})$ (in $\ell ^{\infty }(\mathcal {G_D})$).

The distribution of the test statistics (5) as supremum can then be simulated by simulating Brownian bridges with the covariance function (7) as high-dimensional multivariate Gaussian vectors. The necessary discretisation of the function space $\mathcal {G_D}$ of indicator functions is simple to realise, by choosing a grid of the support *D*. Then for the covariances (7), expressions $\mathbb {E}_{\boldsymbol{\theta }_0}(g_{x_i,t_i,D})$ (*i=1,2* in the notation of the previous proof and *i* not to be confused with *i* as index of the unit in Sect. 2) and $\mathbb {E}_{\boldsymbol{\theta }_0}(g_{x_1,t_1,D}g_{x_2,t_2,D})$ must be calculated. For the preliminary situation of known $\boldsymbol{\theta }_0$ and *n* those two expressions can be calculated with the model specified in Sect. 2, but are left out here. The necessary amendments for the realistic test statistics (6) with the unknown $\boldsymbol{\theta }_0$ and *n* are now discussed. (Appendix F will list the final expectations and an algorithm summing up the approximating steps will be provided in Sect. 4.4.)

### Distribution when $\boldsymbol{\theta }_0$ is unknown but *n* is known

The formula $\sqrt{n}( \mathbb {P}_n(g_{x,t,D})- \mathbb {E}_{\hat{\boldsymbol{\theta }}_n}(g_{x,t,D}))$ (denoted as $\textcircled {1}$) represents the empirical process in which the parameter $\boldsymbol{\theta }_0$ is estimated, and *n* is considered as known (a situation also considered by Shen 2014). Its convergence in distribution follows from the Donsker property in Corollary 1 and Assumptions (A3)-(A5). In order to apply the functional delta method, the representation of the Fréchet derivative for $\boldsymbol{\theta }\mapsto \mathbb {E}_{\boldsymbol{\theta }}$, as a map from *Θ * to $\ell ^{\infty }(\mathcal {G}_D)$ can be specified with Assumption (A2) since the set of functions $\mathcal {G}_D$ is limited to those of the form $1\!\!1_{[0,x]\times [0,t]}1\!\!1_D$ for *(x,t) ∈ S* (with details in Appendix A.1). Explicit representations for two models will follow in Sect. 5.

#### Corollary 2

Under (A2) and $H_0$, the map $\boldsymbol{\theta }\in \Theta \mapsto \mathbb {E}_{\boldsymbol{\theta }} \in \ell ^{\infty }(\mathcal {G}_D)$ has as represent of the Fréchet derivative (at $\boldsymbol{\theta }_0$) $\mathbb {E}_{\boldsymbol{\theta }_0}'(g_{x,t,D}):=\int _{[0,x]\times [0,t]\cap D} \dot{f}_{\boldsymbol{\theta }_0}(\tilde{x},\tilde{t}) \,\textrm{d}(\tilde{x}, \tilde{t})$.

Note that $\dot{g}_{\boldsymbol{\theta }_0}$ is short for $\dot{g}_{\boldsymbol{\theta }}|_{\boldsymbol{\theta }= \boldsymbol{\theta }_0}$. Estimating the parameter causes the limit process of term $\textcircled {1}$ in (10) to no longer be a Brownian bridge process, but it remains Gaussian (van der Vaart 1998, Theorem 19.23). The limit process is derived by decomposing $\textcircled {1}$ once more, and then using the asymptotic linearity (A4) of the estimating sequence. (We will show the asymptotic linearity for our specific models in Sect. 5.) The distribution is summarized in Table 1 as the second line and compared to the situation of both $\boldsymbol{\theta }_0$ and *n* being known. A short calculation using Assumption (A2) yields $\mathbb {E}_{\boldsymbol{\theta }_0}'=\dot{\mathbb {E}}_{\boldsymbol{\theta }_0}$, considered as map $\boldsymbol{\theta }\in \Theta \mapsto \mathbb {E}_{\boldsymbol{\theta }}(g_{x,t,D}) \in \mathbb {R}$, pointwise in $g_{x,t,D}$, and simplifies the representation of the limit process.

**Table 1:** Asymptotic distributions of the test statistic (5) before taking supremum for increasing unavailability of $\boldsymbol{\theta }_0$ (simplifying to $\theta _0$ for independence) and *n*: For first line, results from Sect. 4.1 are sufficient, results for third line are omitted in this paper and results for the fourth line, i.e. test statistics (8), will be given in Sect. 4.3

	Gaussian process representation
$\boldsymbol{\theta }_0,n$	$\mathbb {B}_{\mathbb {P}_{\boldsymbol{\theta }_0}}$
$\hat{\boldsymbol{\theta }}_n, n$	$\mathbb {B}_{\mathbb {P}_{\boldsymbol{\theta }_0}}-\mathbb {B}_{\mathbb {P}_{\boldsymbol{\theta }_0}}( \phi _{\boldsymbol{\theta }_0}) \cdot \mathbb {\dot{E}}_{\boldsymbol{\theta }_0}$
$\boldsymbol{\theta }_0, \hat{n}$	$\mathbb {G}^{1\!\!1}_{\mathbb {P}_{\boldsymbol{\theta }_0}}:= \mathbb {B}_{\mathbb {P}_{\boldsymbol{\theta }_0}} -\mathbb {B}_{\mathbb {P}_{\boldsymbol{\theta }_0}}(1\!\!1_{D}) \cdot \mathbb {E}_{\boldsymbol{\theta }_0}/\alpha _{\boldsymbol{\theta }_0}$
$\hat{\boldsymbol{\theta }}_n, \hat{n}$	$\mathbb {H}_{\mathbb {P}_{\boldsymbol{\theta }_0}}=\mathbb {G}^{1\!\!1}_{\mathbb {P}_{\boldsymbol{\theta }_0}}+\mathbb {B}_{\mathbb {P}_{\boldsymbol{\theta }_0}}(\phi _{\boldsymbol{\theta }_0}) \cdot \left[ \frac{\dot{\alpha }_{\boldsymbol{\theta }_0}}{\alpha _{\boldsymbol{\theta }_0}}\mathbb {E}_{\boldsymbol{\theta }_0}-\dot{\mathbb {E}}_{\boldsymbol{\theta }_0}\right]$

### Distribution when $\boldsymbol{\theta }_0$ and *n* are unknown

Write the test statistic (6) now in a functional-valued notation. We set

8 $$\begin{aligned} \mathbb {T}(g_{x,t,D}):=\frac{\sqrt{\alpha _{\hat{\boldsymbol{\theta }}_n}}}{\sqrt{M_n}}n\mathbb {P}_n(g_{x,t,D})-\frac{\sqrt{M_n}}{\sqrt{\alpha _{\hat{\boldsymbol{\theta }}_n}}}\mathbb {E}_{\hat{\boldsymbol{\theta }}_n}(g_{x,t,D}), \end{aligned}$$

which is to be maximized in absolute terms, i.e. (6) is equal to

9 $$\begin{aligned} \sup _{g_{x,t,D} \in \mathcal {G}_D} \left| \mathbb {T}(g_{x,t,D}) \right| . \end{aligned}$$

Note that $n\mathbb {P}_n(g_{x,t,D})$ does not depend on *n*. In a first step, we determine the asymptotic behaviour of $\mathbb {T}(g_{x,t,D})$ by decomposing it into several sub-problems, that are analysed separately.

When we add to Eq. (8) the terms

$$\begin{aligned} \pm \sqrt{n}\left( \mathbb {P}_n(g_{x,t,D})- \mathbb {E}_{\hat{\boldsymbol{\theta }}_n}(g_{x,t,D})\right) \pm \sqrt{\alpha _{\boldsymbol{\theta }_0}} \left( \frac{n}{\sqrt{M_n}} \mathbb {P}_n(g_{x,t,D})-\frac{\sqrt{n}}{\sqrt{\alpha _{\hat{\boldsymbol{\theta }}_n}}}\mathbb {E}_{\hat{\boldsymbol{\theta }}_n}(g_{x,t,D})\right) , \end{aligned}$$

the following representation results (after factoring out $\mathbb {P}_n(g_{x,t,D})$ and $\mathbb {E}_{\hat{\boldsymbol{\theta }}_n}(g_{x,t,D})$):

$$\begin{aligned}&\sqrt{n}\left( \mathbb {P}_n(g_{x,t,D})- \mathbb {E}_{\hat{\boldsymbol{\theta }}_n}(g_{x,t,D})\right) \\&+\frac{n}{\sqrt{M_n}} \mathbb {P}_n(g_{x,t,D}) \left[ \left( \sqrt{\alpha _{\hat{\boldsymbol{\theta }}_n}}-\sqrt{\alpha _{\boldsymbol{\theta }_0}}\right) - \left( \frac{\sqrt{M_n}}{\sqrt{n}}-\sqrt{\alpha _{\boldsymbol{\theta }_0}}\right) \right] \\&+ \frac{\mathbb {E}_{\hat{\boldsymbol{\theta }}_n}(g_{x,t,D})}{\sqrt{\alpha _{\hat{\boldsymbol{\theta }}_n}}} \left[ \left( \sqrt{n} \sqrt{\alpha _{\hat{\boldsymbol{\theta }}_n}}-\sqrt{n} \sqrt{\alpha _{\boldsymbol{\theta }_0}}\right) - \left( \sqrt{M_n}-\sqrt{n} \sqrt{\alpha _{\boldsymbol{\theta }_0}}\right) \right] \end{aligned}$$

Another factoring out in the last two summands of the preceding display leads to

10 $$\begin{aligned} \begin{aligned}&{\textcircled {1}} + \left[ \underbrace{\sqrt{n}\left( \sqrt{\alpha _{\hat{\boldsymbol{\theta }}_n}}-\sqrt{\alpha _{\boldsymbol{\theta }_0}}\right) }_{{\textcircled {2}}}-\underbrace{\left( \sqrt{M_n}-{\sqrt{n}}\sqrt{\alpha _{\boldsymbol{\theta }_0}}\right) }_{{\textcircled {3}}}\right] \\&\cdot \underbrace{\left[ \frac{\sqrt{n}}{\sqrt{M_n}} \mathbb {P}_n(g_{x,t,D})+ \frac{\mathbb {E}_{\hat{\boldsymbol{\theta }}_n}(g_{x,t,D})}{\sqrt{\alpha _{\hat{\boldsymbol{\theta }}_n}}}\right] }_{{\textcircled {4}}}. \end{aligned} \end{aligned}$$

With the mentioned asymptotic linearity, we can also rewrite term $\textcircled {2}$ of Eq. (10). (The proof can be found in Appendix A.2.)

#### Lemma 1

With Assumptions (A1)-(A5) and under $H_0$, the asymptotic linearity

11 $$\begin{aligned} \sqrt{n}( \sqrt{\alpha _{\hat{\boldsymbol{\theta }}_n}}- \sqrt{\alpha _{\boldsymbol{\theta }_0}})=\frac{1}{\sqrt{n}} \sum _{i=1}^n \phi _{\boldsymbol{\theta }_0}(X_i,T_i)^{\text {T}}\cdot \frac{\dot{\alpha }_{\boldsymbol{\theta }_0}}{2\sqrt{\alpha _{\boldsymbol{\theta }_0}}}+o_{\mathbb {P}_{\boldsymbol{\theta }_0}}(1) \end{aligned}$$

holds.

The convergence in distribution of the univariate sequence in term $\textcircled {3}$ of Eq. (10) can be obtained with the delta method (see e.g. van der Vaart 1998, Sect. 3.1). Note that, because $M_n$ is a binomial random variable, $\sqrt{n}\left( \frac{1}{n}M_n-\alpha _{\boldsymbol{\theta }_0}\right) \rightarrow \mathcal {N}\left( 0, \alpha _{\boldsymbol{\theta }_0}(1-\alpha _{\boldsymbol{\theta }_0})\right)$ as $n \rightarrow \infty$. Thus, we select $x \mapsto \sqrt{x}$ as a function for the delta method, which is differentiable with $(2\sqrt{x})^{-1}$, *x>0*, so that

12 $$\begin{aligned} \sqrt{n}\left( \sqrt{M_n/n}-\sqrt{\alpha _{\boldsymbol{\theta }_0}}\right) \rightarrow \mathcal {N}(0,(1-\alpha _{\boldsymbol{\theta }_0})/4). \end{aligned}$$

The following lemma provides an approximation of term $\textcircled {4}$, the last remaining in Eq. (10). The proof is provided in Appendix A.3.

#### Lemma 2

Under Assumptions (A1)-(A3) and $H_0$, it is

$$\begin{aligned} \left\| \frac{\sqrt{n}}{\sqrt{M_n}} \mathbb {P}_n(g_{x,t,D})+ \frac{\mathbb {E}_{\hat{\boldsymbol{\theta }}_n}(g_{x,t,D})}{\sqrt{\alpha _{\hat{\boldsymbol{\theta }}_n}}} - 2 \cdot \frac{\mathbb {E}_{\boldsymbol{\theta }_0}(g_{x,t,D})}{\sqrt{\alpha _{\boldsymbol{\theta }_0}}} \right\| _{\mathcal {G}_D}\rightarrow 0 \end{aligned}$$

in probability $\mathbb {P}_{\boldsymbol{\theta }_0}$ as $n \rightarrow \infty$.

We now combine the four series in decomposition (10) of (8).

#### Theorem 1

Suppose that (A1) - (A5) hold, then the sequence

$$\begin{aligned} \left\{ \frac{\sqrt{\alpha _{\hat{\boldsymbol{\theta }}_n}}}{\sqrt{M_n}}n\mathbb {P}_n(g_{x,t,D})-\frac{\sqrt{M_n}}{\sqrt{\alpha _{\hat{\boldsymbol{\theta }}_n}}}\mathbb {E}_{\hat{\boldsymbol{\theta }}_n}(g_{x,t,D}): g_{x,t,D} \in \mathcal {G}_D\right\} \end{aligned}$$

converges in distribution in $\ell ^{\infty }(\mathcal {G}_D)$ under $H_0$ to the Gaussian process with values

$$\begin{aligned} \mathbb {H}_{\mathbb {P}_{\boldsymbol{\theta }_0}}(g_{x,t,D}):=\,&\mathbb {B}_{\mathbb {P}_{\boldsymbol{\theta }_0}} (g_{x,t,D})-\left( \mathbb {B}_{\mathbb {P}_{\boldsymbol{\theta }_0}}(\phi _{\boldsymbol{\theta }_0,1}),\mathbb {B}_{\mathbb {P}_{\boldsymbol{\theta }_0}}(\phi _{\boldsymbol{\theta }_0,2})\right) \cdot \dot{\mathbb {E}}_{\boldsymbol{\theta }_0}(g_{x,t,D})\\&+\left[ \left( \mathbb {B}_{\mathbb {P}_{\boldsymbol{\theta }_0}}( \phi _{\boldsymbol{\theta }_0,1}), \mathbb {B}_{\mathbb {P}_{\boldsymbol{\theta }_0}}( \phi _{\boldsymbol{\theta }_0,2})\right) \cdot \dot{\alpha }_{\boldsymbol{\theta }_0} - \mathbb {B}_{\mathbb {P}_{\boldsymbol{\theta }_0}}(1\!\!1_D) \right] \cdot \frac{\mathbb {E}_{\boldsymbol{\theta }_0}(g_{x,t,D})}{\alpha _{\boldsymbol{\theta }_0}} \end{aligned}$$

with $$g_{x,t,D} \in \mathcal {G}_D$$.

Before the proof, we would like to stress that the practically necessary covariance function will be given in Sect. 4.4 in a general form and for two specific models in Sects. 5.1 and 5.2. The detailed proof is in Appendix B. In brief, in view of (10), Corollary 1 and Corollary 2 are applied to term $\textcircled {1}$, Lemma 1 is applied to term $\textcircled {2}$, term $\textcircled {3}$ is treated as above and Lemma 2 is applied to term $\textcircled {4}$. Then it is used that the Donsker property of two classes extends to their union, the functional delta method as well as the continuous mapping theorem for metric spaces are applied.

Table 1 lists the distribution and also compares it to the situation of known $\boldsymbol{\theta }_0$ and estimated *n* (with derivation suppressed here) in order to show the increasing complexity.

The corresponding convergence of the sequence in the supremum norm $\Vert \cdot \Vert _{\mathcal {G}_D}$, i.e. the convergence of the modified Kolmogorov-Smirnov test statistic (9) (or equivalently (6)), follows from the ordinary continuous mapping theorem.

### Computation of critical value

The distribution of $\mathbb {H}_{\mathbb {P}_{\boldsymbol{\theta }_0}}$ has been derived under hypothesis in Theorem 1. We now determine that of $\Vert \mathbb {H}_{\mathbb {P}_{\boldsymbol{\theta }_0}} \Vert _{\mathcal {G}_D}$, which can be simulated using the involved Gaussian processes. The Gaussian process $\mathbb {H}_{\mathbb {P}_{\boldsymbol{\theta }_0}}$ has zero mean and Appendix D derives as a general representation of the covariance between $\mathbb {H}_{\mathbb {P}_{\boldsymbol{\theta }_0}}(g_{x_1,t_1,D})$ and $\mathbb {H}_{\mathbb {P}_{\boldsymbol{\theta }_0}}(g_{x_2,t_2,D})$:

13 $$\begin{aligned} \begin{aligned}&\mathbb {E}_{\boldsymbol{\theta }_0}(g_{x_1,t_1,D}g_{x_2,t_2,D})-\mathbb {E}_{\boldsymbol{\theta }_0}(g_{x_1,t_1,D})\mathbb {E}_{\boldsymbol{\theta }_0}(g_{x_2,t_2,D})/\alpha _{\boldsymbol{\theta }_0}+K_2^{\text {T}} \mathbb {E}_{\boldsymbol{\theta }_0}(g_{x_1,t_1,D}\phi _{\boldsymbol{\theta }_0})\\&\quad +K_1^{\text {T}}\mathbb {E}_{\boldsymbol{\theta }_0}(g_{x_2,t_2,D} \phi _{\boldsymbol{\theta }_0})+K_1^{\text {T}} \mathbb {E}_{\boldsymbol{\theta }_0}(\phi _{\boldsymbol{\theta }_0}\phi _{\boldsymbol{\theta }_0}^{\text {T}})K_2, \end{aligned} \end{aligned}$$

where $K_i:=(K_{i,\theta _0}, K_{i, \vartheta _0})^{\text {T}}$ and $K_{i,j}:=\left( \frac{\dot{\alpha }_{j}}{\alpha _{\boldsymbol{\theta }_0}}\mathbb {E}_{\boldsymbol{\theta }_0}g_{x_i,t_i,D}-\dot{\mathbb {E}}_{j}g_{x_i,t_i,D}\right)$, for *i=1,2* and $j \in \{\theta _0, \vartheta _0\}$. (Note that *i* and *j* here are not indicators for units as in Sect. 2.) For the two different copulas, Sect. 5 will provide more specific representations. Therefore, the expectations of the indicator function $g_{x_i,t_i,D}$ and of the score function $\phi _{\boldsymbol{\theta }_0}$, as well as the derivative $\dot{\mathbb {E}}_{j}$ need to be calculated. Those will be given in closed form in Appendix F. (For calculating $\mathbb {E}_{\theta _0}(g_{\min (x_1, x_2),\min (t_1,t_2),D})$, we simply need to determine the minimum values and insert it into the formula for $\mathbb {E}_{\theta _0}(g_{x_i,t_i,D})$.) Even though $\boldsymbol{\theta }_0$ is not assumed to be known in derivation of the test, as usual, the asymptotic test distribution still depends on $\boldsymbol{\theta }_0$, but can be replaced by $\hat{\boldsymbol{\theta }}_n$ by use of the ordinary continuous mapping theorem. The commonly used Cholesky decomposition for random field generation (see e.g. Liu et al. 2019) will be simplified when applied to simulate the distribution of $\Vert \mathbb {H}_{\mathbb {P}_{\hat{\boldsymbol{\theta }}_n}} \Vert _{\mathcal {G}_D}$, because the support is compact.

**Algorithm 2:**
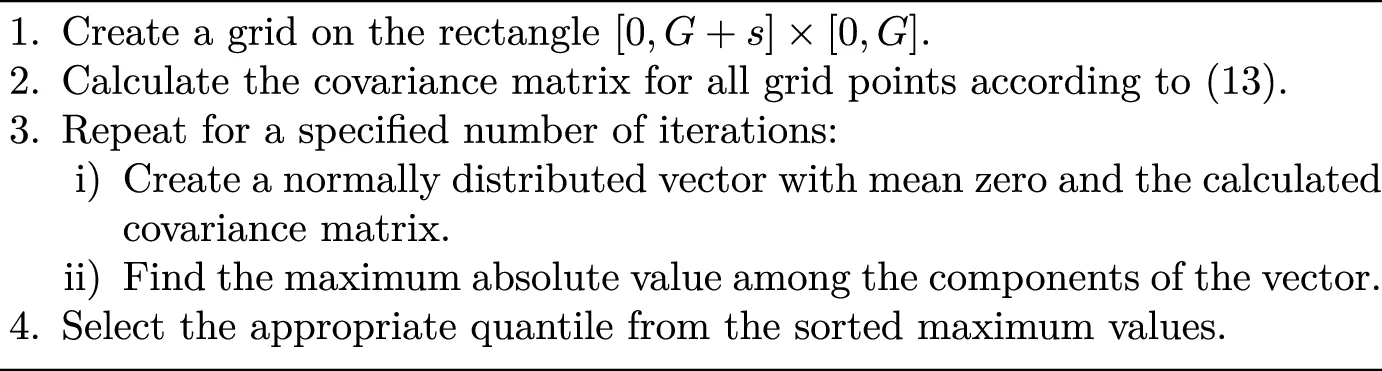
Computing the critical value of (6)

As strategy to make the computation feasible, the supremum of the limiting Gaussian process can be approximated on a finite grid, which reduces the infinite-dimensional problem to a tractable finite-dimensional setting. A key aspect of the computational implementation is the efficient evaluation of integrals over the truncation region. In particular, substantial computational savings can be achieved by exploiting the geometric structure of the domain. In our setting, the truncation region can be decomposed into simpler geometric shapes, namely rectangles and triangles, which allows us to reduce the problem to a finite number of cases (five in total). This decomposition enables a case-wise analytical treatment of the integrals. Whenever the functional form of the copula permits, the resulting (double) integrals can be evaluated in closed form, thereby avoiding costly numerical integration and significantly improving computational efficiency. The covariance matrix can be computed once and reused for all Monte Carlo simulations. Critical values are obtained via Monte Carlo simulation of a multivariate normal distribution with the derived covariance structure. The implementation relies on vectorized operations and avoids redundant evaluations of integrals and model components. While the covariance matrix scales quadratically in the grid size, a well-chosen discretization level can provide a good balance between computational feasibility and approximation accuracy.

## Testing the exponential distribution

Suppose one wants to test whether the hazard is homogenous, i.e. whether the lifespan *X* has an exponential density $f_{\theta }^X(x)=\theta \text {exp}(- \theta x) \; (\theta > 0)$ in hypothesis (3). Mainly for the ease of display, we will assume *T* to be uniformly distributed. For the dependence we consider two cases, independence and a Farlie-Gumbel-Morgenstern copula. For calculating the two test statistics with Algorithm 1, point estimates for the parameters are needed. For the critical values, the Assumptions (A1)-(A5) considering the models and the sampling design must hold in order to apply Theorem 1. Furthermore, the two model-specific versions of the covariance function (13) must be derived in order to apply Algorithm 2.

### Independent truncation: product copula

Note that the product copula $C^{\text {ind}}(u,v):=u \cdot v$ does not depend on a parameter, so that under hypothesis $F_{\theta }=F^X_{\theta } \cdot F^T$ with $\Theta =(\varepsilon , 1/\varepsilon )$.

Assumption (A1) results from $\alpha _{\theta } \in (0,1)$ Weißbach and Wied (2022,  Eq. (1)) where *0< G < ∞ *, *s>0*, so that The integrability and continuous differentiability of the density $f_{\theta }(x,t)=\theta e^{-\theta x}/G$ are consequences of the corresponding properties of the exponential function. The interchangeability of integrating for (*x*, *t*) and differentiating for *θ * can be demonstrated through direct calculations. The *Z*-estimator is defined as the zero of the criterion function given in Weißbach and Wied (2022,  Eq. (5)), where the score function involves the derivative $\dot{\alpha }_{\theta }$ of $\alpha _{\theta }$ and is defined as

14 $$\begin{aligned} \psi _{\theta }(X_i,T_i)=1\!\!1_D(X_i,T_i)\left[ X_i-\frac{1}{\theta }+\frac{\dot{\alpha }_{\theta }}{\alpha _{\theta }}\right] . \end{aligned}$$

Weißbach and Wied (2022, Theorems 1 and 2) show that Assumptions (A3) and (A4) hold. Note that the relation $\phi _{\theta _0}=-(\mathbb {E}_{\theta _0} [ \dot{\psi }_{\theta _0}])^{-1}\psi _{\theta _0}$ and Assumption (A5) follows from Weißbach and Wied (2022, Lemmas 2 and 3). Especially, it is $\sup _{(x,t) \in D} \vert x - \theta _0^{-1}+\dot{\alpha }_{\theta _0} \alpha _{\theta _0}^{-1} \vert < \infty$ and $\dot{\mathbb {E}}_{\theta _0}[\psi _{\theta _0}]>0$ and thus $\sup _{(x,t) \in S} \vert \phi _{\theta _0}(x,t)\vert < \infty$. Therefore, Theorem 1 applies and due to the information matrix equality (see Toparkus and Weißbach 2025, Eq. (11)) and the relation $\psi _{\theta _0}=\dot{f}_{\theta _0}/f_{\theta _0}-\dot{\alpha }_{\theta _0}/\alpha _{\theta _0}$ on *D*, the covariance function simplifies to

15 $$\begin{aligned} \begin{aligned}&\mathbb {E}_{\theta _0}(g_{\min (x_1,x_2),\min (t_1,t_2),D})- \mathbb {E}_{\theta _0}(g_{x_1,t_1,D}) \mathbb {E}_{\theta _0}(g_{x_2,t_2,D})/\alpha _{\theta _0}\\&+(\mathbb {E}_{\theta _0} [ \dot{\psi }_{\theta _0}])^{-1} \cdot \mathbb {\dot{E}}_{\theta _0}(g_{x_1,t_1,D}) \mathbb {\dot{E}}_{\theta _0}(g_{x_2,t_2,D})\\&-(\mathbb {E}_{\theta _0} [ \dot{\psi }_{\theta _0}])^{-1} \cdot \frac{\dot{\alpha }_{\theta _0}}{\alpha _{\theta _0}}\cdot \left[ \mathbb {\dot{E}}_{\theta _0}(g_{x_2,t_2,D}) \mathbb {E}_{\theta _0}(g_{x_1,t_1,D})+\mathbb {\dot{E}}_{\theta _0}(g_{x_1,t_1,D}) \mathbb {E}_{\theta _0}(g_{x_2,t_2,D})\right] \\&+(\mathbb {E}_{\theta _0} [ \dot{\psi }_{\theta _0}])^{-1} \cdot \frac{\dot{\alpha }_{\theta _0}^2}{\alpha _{\theta _0}^2}\cdot \mathbb {E}_{\theta _0}(g_{x_1,t_1,D})\mathbb {E}_{\theta _0}(g_{x_2,t_2,D}). \end{aligned} \end{aligned}$$

Its elements are given in Appendix F.1. In order to distinguish the two tests in the applications later Sect. 7, we will use the superscript “$\text {ind}$”, e.g. for *C* and the selection probability. By contrast, *θ * has the same meaning for independence and dependence. However, its estimation differs, of course, and the superscript “$\text {ind}$” for $\hat{\theta }_n$ and $\alpha _{\hat{\theta }_n}$ will indicate the first test.

### Dependent truncation: Farlie-Gumbel-Morgenstern copula

Only few restrictions for dependence by one additional parameter are implied by the Farlie-Gumbel-Morgenstern (FGM) copula,

$$\begin{aligned} C^{\text {FGM}}_{\vartheta }(u,v):=u v [1+\vartheta (1-u)(1-v)], \, (\vartheta \in [-1,1]), \end{aligned}$$

where codomain of the parameter *ϑ * enables positive *(ϑ >0)* and negative dependence *(ϑ <0)*, as well as independence *(ϑ =0)*. (In order to distinguish between the symbols in Sect. 7, we already use the superscript $\text {FGM}$ in this section.) For modelling dependence between lifespan and truncation age, as another candidate, the Gumbel-Barnett copula (see e.g. Toparkus and Weißbach 2025) can only be applied if the direction of nonstationary is known to be “upwards”, as in human demography. Another advantage over the Gumbel-Barnett copula (but also over the Clayton-copula) is analytical tractability in order to derive the observation probability (with derivative), the score function (for both see Sect. 5.2) and the asymptotic covariance function (see Appendix F.2). And it should be added that the FGM copula does not model tail dependence. As disadvantage could be seen that dependence, measured with Kendall’s tau, cannot be stronger than $\tau _{\vartheta }^{\text {FGM}}=2\vartheta /9$, which however is typically sufficient in economic and social sciences, and especially in our data example. The density of $(X_i,T_i)^{\text {T}}$ results in

$$\begin{aligned} f^{\text {FGM}}_{\boldsymbol{\theta }}(x,t)= \frac{\theta }{G}e^{-\theta x} \left[ 1+\vartheta (2e^{-\theta x} -1) \left( 1-\frac{2t}{G}\right) \right] , \end{aligned}$$

for *x>0* and *0<t<G*, and is zero elsewhere. Hence, the parameter space is two-dimensional with $\Theta =(\varepsilon _{\theta }, 1/\varepsilon _{\theta })\times (\varepsilon _{\vartheta }-1,1-\varepsilon _{\vartheta })$. For the goodness-of-fit test of the hypotheses (3), it is now $F_{\boldsymbol{\theta }}=C^{\text {FGM}}_{\vartheta }(F^X_{\theta },F^T)$. Again, we need to verify the Assumptions (A1)-(A5), now for the FGM copula.

For the Gumbel-Barnett copula, Assumptions (A1)-(A5) are proven in Toparkus and Weißbach (2025, Lemmas 1,3,4, Theorems 1,2). That is, in order to prove Assumption (A1) for the FGM copula, consider the observation probability (see Toparkus and Weißbach, 2025 after Eq. (2)), which can be represented as $\alpha _{\boldsymbol{\theta }}^{\text {FGM}} = \alpha _{\theta }^{\text {ind}} - G^{-1} \vartheta \beta _{\theta } + G^{-2} \vartheta \gamma _{\theta }$, where $\beta _{\theta }:= ( - \theta ^{-1} (e^{-\theta s} - 1)(e^{-\theta G} + 1) + (2\theta )^{-1} (e^{-2\theta s} - 1)(e^{-2\theta G} + 1))$ and $\gamma _{\theta }:= ( \theta ^{-2} (1 - e^{-\theta s})(1 - e^{-\theta G}) - (2\theta )^{-2} (1 - e^{-2\theta s})(1 - e^{-2\theta G}))$. It can proven to be in (0, 1).

In order to see Assumption (A2), note that the integrability and continuous differentiability of the density $f_{\boldsymbol{\theta }}^{\text {FGM}}$ are consequences of the corresponding properties of the exponential function. The interchangeability of integrating for (*x*, *t*) and differentiating for $\boldsymbol{\theta }$ can be demonstrated through straightforward calculations.

The consistency stated in Assumption (A3) can be established using techniques analogous to those applied in the Gumbel–Barnett copula case in Toparkus and Weißbach (2025, Theorem1), starting from the two-dimensional score function $\psi _{\boldsymbol{\theta }}^{\text {FGM}}$ which involves the gradient $\dot{\alpha }_{\boldsymbol{\theta }}^{\text {FGM}}$ of $\alpha _{\boldsymbol{\theta }}^{\text {FGM}}$ (see Appendix E) and has coordinates:

16 $$\begin{aligned} \begin{aligned} \psi _{\boldsymbol{\theta },1}^{\text {FGM}}(X_i,T_i)=1\!\!1_{[T_i,T_i+s]}(X_i)\Bigg [\frac{1}{\theta }-X_i-\frac{2\vartheta X_i e^{-\theta X_i}\left( 1-\frac{2T_i}{G}\right) }{1+\vartheta \left( 2e^{-\theta X_i}-1\right) \left( 1-\frac{2T_i}{G}\right) } \\ -\frac{\dot{\alpha }_{\theta }^{\text {FGM}}}{\alpha _{\boldsymbol{\theta }}^{\text {FGM}}}\Bigg ] \\ \psi _{\boldsymbol{\theta },2}^{\text {FGM}}(X_i,T_i)=1\!\!1_{[T_i,T_i+s]}(X_i)\left[ \frac{\left( 2 e^{-\theta X_i}-1\right) \left( 1-\frac{2T_i}{G}\right) }{1+\vartheta \left( 2e^{-\theta X_i}-1\right) \left( 1-\frac{2T_i}{G}\right) }-\frac{\dot{\alpha }_{\vartheta }^{\text {FGM}}}{\alpha _{\boldsymbol{\theta }}^{\text {FGM}}}\right] \end{aligned} \end{aligned}$$

Note that the FGM copula has a simpler structure than the Gumbel-Barnett copula and simplifies calculations. Assumption (A4), the asymptotic linearity with the relation $\phi _{\boldsymbol{\theta }_0}=-(\mathbb {E}_{\boldsymbol{\theta }_0} [ \dot{\psi }_{\boldsymbol{\theta }_0}])^{-1}\psi _{\boldsymbol{\theta }_0}$, follows similarly as in Toparkus and Weißbach (2025, Theorem 2). On proving Assumption (A5) (see Toparkus and Weißbach 2025, Lemmas 3 and 4). E.g., for verifying $\sup _{(x,t)\in S}\Vert \phi _{\boldsymbol{\theta }_0}(x,t)\Vert <\infty$, note that for the denominators in $\psi _{\boldsymbol{\theta }_0,1}$ and $\psi _{\boldsymbol{\theta }_0,2}$ it is

$$\begin{aligned} \left| 1 + \vartheta _0 (2e^{-\theta _0 x}-1) \left( 1-\frac{2t}{G}\right) \right| \ge \left| 1 - \left| \vartheta _0 (2e^{-\theta _0 x}-1) \left( 1-\frac{2t}{G}\right) \right| \right| \ge 1- \vert \vartheta _0 \vert \end{aligned}$$

on *D* by the reverse triangle inequality. The edge case $\vert \vartheta _0\vert =1$ is not included in the parameter space. The two longer numerators in (16) can be bounded above on *D* by $\left| 2 \vartheta _0 x e^{-\theta _0 x} \left( 1-\frac{2t}{G}\right) \right| \le 2 \vert \vartheta _0\vert (G+s)$ and $\left| (2 e^{-\theta _0 x} -1) \left( 1-\frac{2t}{G}\right) \right| \le 1$.

Thus, Theorem 1 applies, and due to the information matrix equality (see Toparkus and Weißbach, 2025, Eq. (11)) and the relation $\psi _{\boldsymbol{\theta }_0}=\dot{f}_{\boldsymbol{\theta }_0}/f_{\boldsymbol{\theta }_0}-\dot{\alpha }_{\boldsymbol{\theta }_0}/\alpha _{\boldsymbol{\theta }_0}$ on *D*, we can simplify the covariance function to

17 $$\begin{aligned} \begin{aligned}&\mathbb {E}_{\boldsymbol{\theta }_0}(g_{\min (x_1,x_2),\min (t_1,t_2),D})- \mathbb {E}_{\boldsymbol{\theta }_0}(g_{x_1,t_1,D}) \mathbb {E}_{\boldsymbol{\theta }_0}(g_{x_2,t_2,D})/\alpha _{\boldsymbol{\theta }_0}\\&-\mathbb {\dot{E}}_{\boldsymbol{\theta }_0}(g_{x_2,t_2,D})^{\text {T}}\cdot (-\mathbb {E}_{\boldsymbol{\theta }_0} [ \dot{\psi }_{\boldsymbol{\theta }_0}])^{-1} \cdot \mathbb {\dot{E}}_{\boldsymbol{\theta }_0}(g_{x_1,t_1,D}) \\&+\frac{\mathbb {E}_{\boldsymbol{\theta }_0}(g_{x_1,t_1,D})}{\alpha _{\boldsymbol{\theta }_0}} \cdot \mathbb {\dot{E}}_{\boldsymbol{\theta }_0}(g_{x_2,t_2,D})^{\text {T}}\cdot (-\mathbb {E}_{\boldsymbol{\theta }_0} [ \dot{\psi }_{\boldsymbol{\theta }_0}])^{-1} \cdot \dot{\alpha }_{\boldsymbol{\theta }_0}\\&+\frac{\mathbb {E}_{\boldsymbol{\theta }_0}(g_{x_2,t_2,D})}{\alpha _{\boldsymbol{\theta }_0}} \cdot \mathbb {\dot{E}}_{\boldsymbol{\theta }_0}(g_{x_1,t_1,D})^{\text {T}}\cdot (-\mathbb {E}_{\boldsymbol{\theta }_0} [ \dot{\psi }_{\boldsymbol{\theta }_0}])^{-1} \cdot \dot{\alpha }_{\boldsymbol{\theta }_0}\\&-\frac{\mathbb {E}_{\boldsymbol{\theta }_0}(g_{x_1,t_1,D})}{\alpha _{\boldsymbol{\theta }_0}}\frac{\mathbb {E}_{\boldsymbol{\theta }_0}(g_{x_2,t_2,D})}{\alpha _{\boldsymbol{\theta }_0}} \cdot \dot{\alpha }_{\boldsymbol{\theta }_0}^{\text {T}} \cdot (-\mathbb {E}_{\boldsymbol{\theta }_0} [ \dot{\psi }_{\boldsymbol{\theta }_0}])^{-1} \cdot \dot{\alpha }_{\boldsymbol{\theta }_0}. \end{aligned} \end{aligned}$$

The elements are given in Appendix F.2.

## Approximation errors

The derived test relies on some approximations. To start with, the critical value of the test in Algorithm 2 is based on a discrete grid approximation of *S*. And, as a typical aspect of asymptotic statistics, the actual sample size is only finite. In Sect. 7, where the estimated observation probability will be 0.1 (with or without truncation dependence) the $m_n \approx 50{,}000$ observations imply a sample size of $n \approx 500{,}000$. This suggests a high accuracy of the asymptotic critical value. And additionally there will be a large difference between the test statistic (with or without truncation dependence) and the critical value (for levels 5% or 1%), so that the decision in our application is unlikely to be incorrect due to the approximations.

However, we must still keep in mind that, whereas for uni- or multivariate random sequences with a limiting Gaussian distribution the convergence speed is known to be fast, e.g. by the Berry-Esseen bound, the convergence of the function-valued argument in the test statistic (6) may be considerably slower. Additionally, the asymptotic distribution of the test statistic (see Theorem 1), including the covariance function, depends on the true $\boldsymbol{\theta }_0$. In the final test, $\hat{\boldsymbol{\theta }}_n$ must replace $\boldsymbol{\theta }_0$, leading again to an error.

This section compares in particular nominal and actual level and assesses power, under both, truncation dependence and truncation independence. The conditions mimic our population models (see Sect. 5) and the design of the observed data (see Sect. 7.1), with smaller sample sizes. However, before we assess the finite sample properties, we add some algorithmic details, starting with the discrete grid approximation inherent in Algorithm 2. The marginal distributions in $H_0$ will always be the exponentially distributed for *X* and the uniformly for *T*.

### Numerical techniques

#### The critical value

For simulating a random field in Algorithm 2, the possible values for $(x^{\text {obs}},t^{\text {obs}})^{\text {T}}$ are approximated by a two-dimensional discrete (bounded) grid. The influence of the density of discretisation is assessed here only for dimension one. Kolmogorov (1933) derived the distribution of $\Vert \mathbb {B} \Vert _{\infty }$, with $\mathbb {B}$ as Brownian bridge. Numerical values can be found in Miller (1956); Bleymüller et al. (2021). The following algorithm is used to simulate quantiles for differently dense grids and compare them with the quantiles of the continuous Kolmogorov distribution.

**Algorithm 3:**
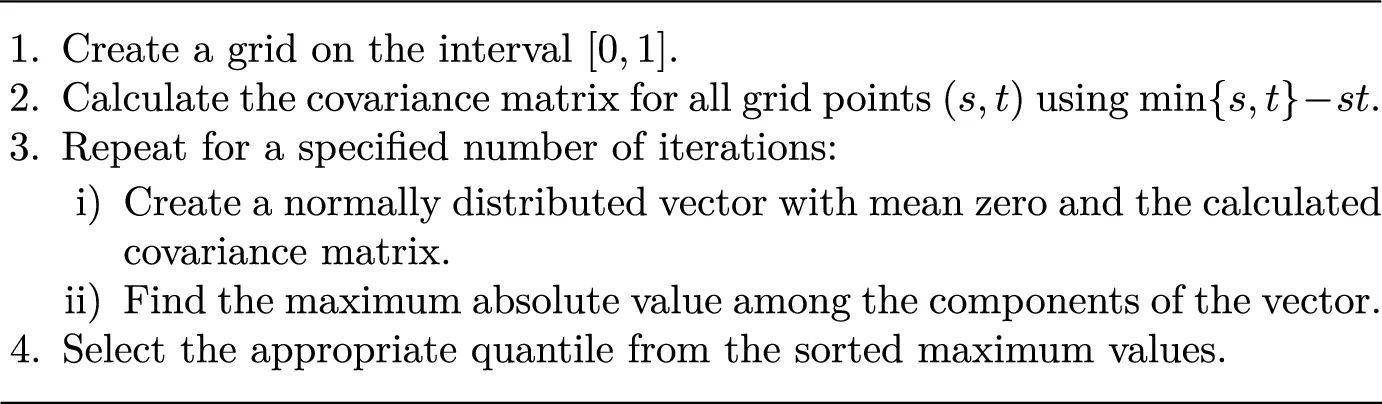
Simulating the distribution of $\Vert \mathbb {B}\Vert _{\infty }$

**Fig. 2:**
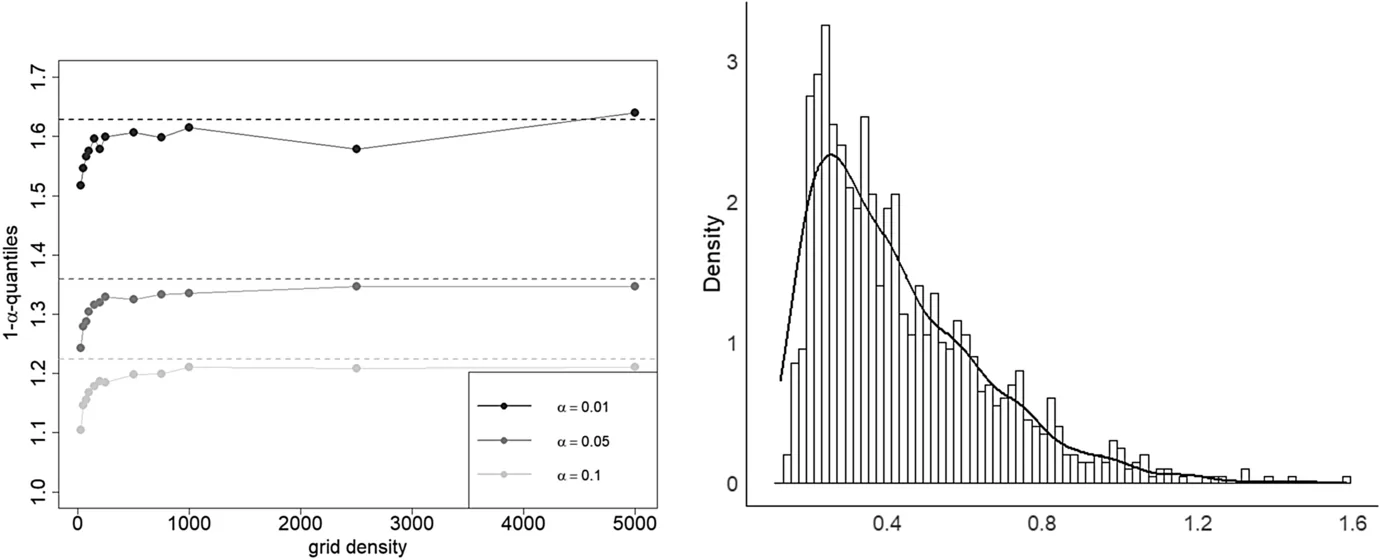
Left: Illustration of the quantiles simulated by Algorithm 3 as a function of test level (*α *) and the grid density, compared to the quantiles of the Kolmogorov distribution (shown as dashed lines), Right: Distribution of test statistic (5) for estimating $\boldsymbol{\theta }_0$ and known *n* of Sect. 4.2: Simulated (histogram, Algorithm 1) versus asymptotic (solid line, Algorithm 2)

The density of the grid varies so that the interval [0, 1] is divided into between 25 and 5,000 sections. In each case, 1,000 realizations of the random variable $\Vert \mathbb {B} \Vert _{\infty }$ are generated and the quantile is selected from them. Figure 2 (left) shows the *(1-α )*-quantiles for $\alpha =0.1,\, 0.05, \,0.01$. The dashed lines indicate the corresponding quantile of the Kolmogorov distribution. A clear convergence can be seen for all three nominal levels.

#### The random variable

With independence assumption between *X* and *T* in $H_0$ (or even $H_1$), the simulation for one sample unit can be performed with the common inversion method.

**Algorithm 4:**
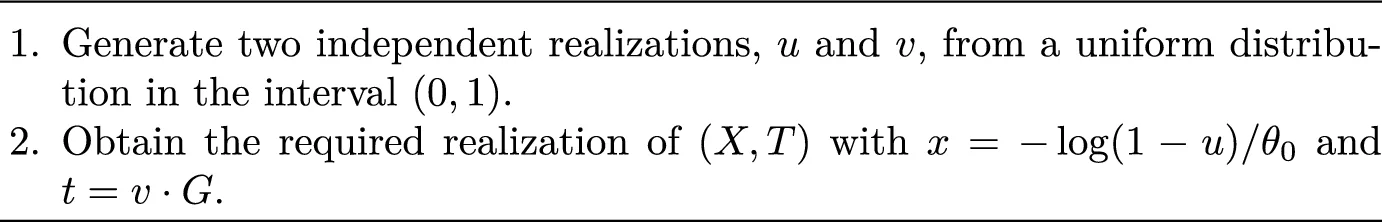
Generation of $(X,T)^{\text {T}}$ (*X* independent of *T*)

Assuming the FGM copula for $(X,T)^{\text {T}}$ (again in either $H_0$ or $H_1$), one further step in the inversion method, the conditional inverse method, uses the partial derivative of the copula,

$$\begin{aligned} c_u^{\text {FGM}}(v):=\partial C_{\vartheta _0}^{\text {FGM}}(u,v) / (\partial u)=v+\vartheta _0 (1-2u)v-\vartheta _0(1-2u)v^2. \end{aligned}$$

**Algorithm 5:**
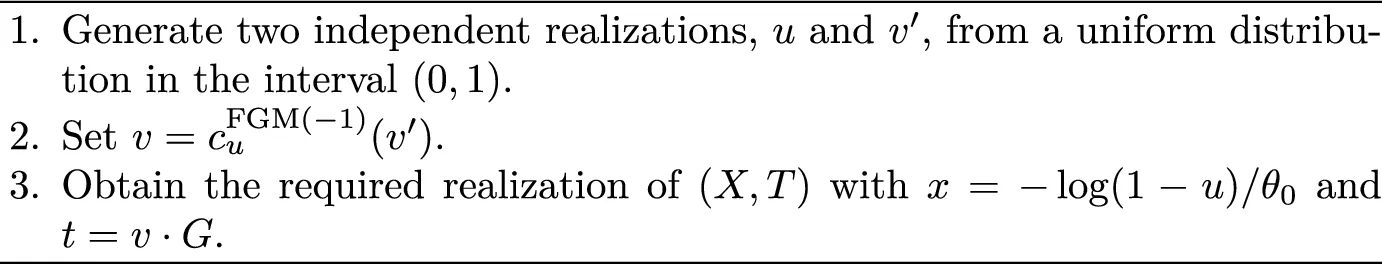
Generation of $(X,T)^{\text {T}}$ (*X* $\text {FGM}$-dependent of *T*)

### Simulation of actual size

We simulate, independent and identically distributed, $(X_1,T_1)^{\text {T}}, \ldots , (X_n,T_n)^{\text {T}}$ for $n=20{,}000$ (and $n=50{,}000$) with the distribution of hypothesis (3). In accordance the the application in Sect. 7, we set as model parameters $\theta _0=0.082$, *G=24* and *s=3*. Thus, after independent truncating the sample elements outside the observation parallelogram *D*, approximately $\alpha _{\theta _0}=0.1$ of the latent sample remain. (For dependence, the second parameter will be chosen below resulting in almost the same observation probability.) In each sample, the one-dimensional point estimator under independence (and the two-dimensional for dependence) is calculated by summing up the score functions in (14), and (16) respectively, followed by and finding its root. The test statistics is subsequently calculated using Algorithm 1. Also for each sample, because the point estimator enters the covariance function (13), is the critical value obtained with Algorithm 2. For this purpose, a grid is placed on the rectangle *[0,G+s]× [0,G]*, with each interval divided into 50 equidistant parts and 1,000 realizations of the Gaussian process from the second line in Table 1 are generated. The absolute suprema of the 1,000 processes are determined and the required empirical quantile is the critical value. The null hypothesis is rejected each time the test statistic exceeds this critical value. The results are given and discussed now for truncation independence and dependence separately.

#### Independent truncation

With independence assumption between $X_i$ and $T_i$ under $H_0$, Algorithm 4 is invoked. Table 2 demonstrates, for several combinations of sample sizes *n* and nominal levels *α *, that the actual level of the test is close to the nominal.

**Table 2:** Rejection rates of the test statistic when the sample size *n* is known: Three nominal levels *α * for independent truncation (“ind”) and FGM-dependent truncation (FGM), three numbers of simulation runs ($n_{sim}$) for *G=24*, *s=3*, $\theta _0=0.082$ (independent) and $\vartheta _0=0.103$ (FGM-dependent), grid density is 50

$n_{sim}$	$n=20{,}000$						$n=50{,}000$					
	*α =0.1*		*α =0.05*		*α =0.01*		*α =0.1*		*α =0.05*		*α =0.01*	
	ind	FGM	ind	FGM	ind	FGM	ind	FGM	ind	FGM	ind	FGM
10	20	0	10	0	0	0	10	1	0	1	0	0
100	9	9	3	4	0	0	11	13	6	8	0	0
1000	11$^2$	10$^9$	5$^2$	5$^4$	1$^3$	1	12	11$^4$	6$^5$	6	1$^5$	1$^6$

Numbers are percentages and position after decimal point are superscript (or zero)

#### FGM-dependent truncation

The FGM-dependence assumed between $X_i$ and $T_i$ under $H_0$, random variable are simulated with Algorithm 5. The only additional population parameter to be chosen, again taken from the application, is $\vartheta _0=0.103$. As in the independent case, the actual level of the test is close to the nominal, as Table 2 demonstrates for several combinations of sample sizes *n*, nominal levels *α *. It is worth noting that the simulations require a substantial amount of computation time, in detail, for $n_{sim}=10, 100, 1000$, runtimes of 22 min, 3.7 h and 37 h, respectively.

Instead of focusing in some nominal levels of a test, we may also use Algorithm 2 (grid density of 50) to display the entire asymptotic distribution (Fig. 2 (right, solid line)). This can be compared with the finite sample distribution (for *n=20,000*), as computable from Algorithm 1), from $n_{sim}=1,000$ samples (Fig. 2 (right, histogram)). The fit appears to be good.

Table 3 shows another simulation that assesses the different rejection rates for different values of $\vartheta _0$. For increasing negative dependence between *X* and *T* the test rejects too often, for increasing positive dependence it rejects too rarely. One could say that it is most reliable for small dependency, as in our example.

**Table 3:** Actual simulated test levels for different dependence strengths $\vartheta _0$ (sample size *n=20.000*, $n_{sim}=100$, grid density 50)

*α * $\vartheta _0$	-1	−0.8	−0.6	−0.4	−0.2	0	0.2	0.4	0.6	0.8	1
0.1	13	14	10	6	7	8	6	8	5	8	3
0.05	4	10	6	2	4	4	3	1	4	3	1
0.01	1	0	0	0	0	0	0	0	1	0	0

A third simulation assesses different truncation severities. Intuitively, estimation must become easier with more data. This also extends, for given latent sample size *n*, for further observations $m_n$. Those are governed by the probability of observation (2). The probability, and with it the number of observations, increases surely in *s*. The influence of *G* is not monotonous on $m_n$. For varying *G* and *s* (centered around the conditions of the application), the actual size (for three nominal levels) are listed in Table 4. Each run needs 3.5−4.5 h on average. The increase of the estimation accuracy with increasing *s* is visible, and also larger values of *G* seem to increase the accuracy.

**Table 4:** Actual simulated test levels for different probabilities of observation due to difference in study length *s* (and population width *G*), $\theta _0=0.082$, $\vartheta _0=0.10256$, $n=20{,}000$, $n_{sim}=100$, grid density 50

G s	*α =0.1*				*α =0.05*				*α =0.01*			
	1	2	3	4	1	2	3	4	1	2	3	4
12	18	14	16	15	13	10	11	12	13	8	6	4
24	11	13	9	6	3	5	4	4	0	1	0	0
30	8	9	10	9	3	6	6	3	0	2	3	1

### Simulation of actual power

As long as not stated otherwise, the simulations are specified as in Sect. 6.2.

#### Independent truncation

**Fig. 3:**
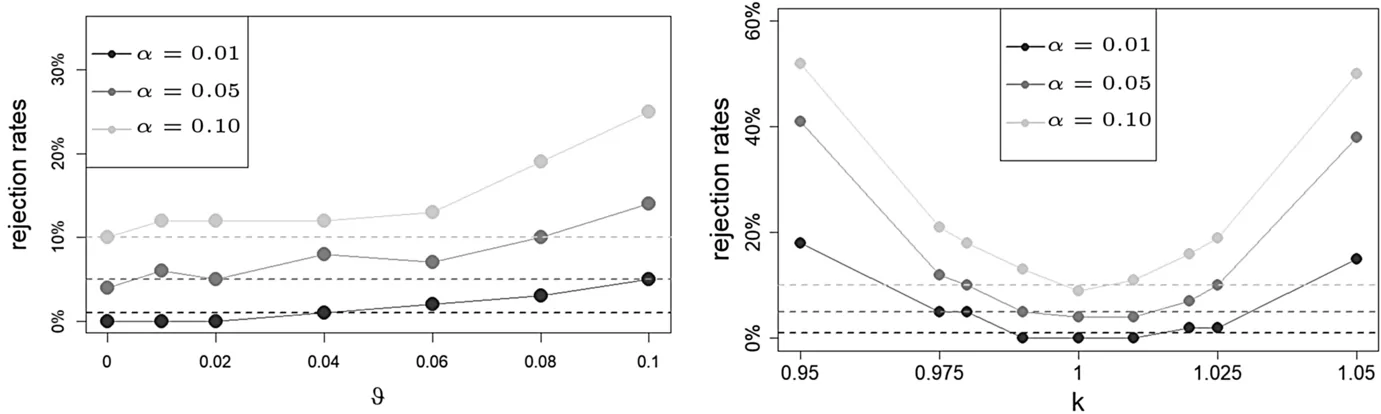
Power of KS-test against $H_0$ (3): $H_0$ with independent truncation and $H_1$ with increasing FGM dependency ($\vartheta _0=0$ is independence) (left), $H_0$ with FGM-dependent truncation and exponential *X*, $H_1$ with Weibull distributed lifespan (*k=1* is exponential distribution) (right) ($n_{sim} = 100$, grid density 50, nominal levels $\alpha =1\%, 5\%, 10\%)$

One possibility to simulate the power of the derived KS test is to use the null hypothesis (3) under independent truncation, i.e. with $C_{\vartheta } = C^{\text {ind}}$ of Sect. 5.1. The sample units $(X_i,T_i)^{\text {T}}$ however will be simulated with truncation FGM dependence, i.e. with Algorithm 5. Than $\vartheta _0=0$ is the null hypothesis and $\vartheta _0 > 0$ is the alternative hypothesis $H_1$ and we use small dependency of $\vartheta _0 \in \{0.01,0.02, 0.04, 0.06, 0.08, 0.1\}$. The number of simulation loops is set to $n_{sim} = 100$ and the grid density to 50. Even for data with low dependency, Fig. 3 (left) shows considerable increase of the rejection rate over that under $H_0$ and the expected monotonous behaviour.

#### FGM-dependent truncation

Consider the FGM copula as part of $H_0$. If the $(X_i,T_i)^{\text {T}}$’s are simulated with a Gumbel-Barnett copula (see e.g. Toparkus and Weißbach, 2025, Assumption (A3)), the rejection rates are power estimates. For a fair comparison, consider Kendall’s *τ *. The Gumbel-Barnett copula only models negative dependence and for the FGM copula with negative dependence it is $\tau \in [-2/9, 0]$. For the simulation we determine the second parameters $\vartheta ^{\text {FGM}}$ and $\vartheta ^{\text {GB}}$, respectively, (additional to location *θ *) for both copulas so that Kendall’s *τ * is equal. In general *τ * can range between −1 and 1. As Table 5 shows, the test is able to distinguish the Gumbel-Barnett from the FGM copula starting from a Kendall’s *τ * of −0.1.

**Table 5:** Power for distinguishing the Farlie-Gumbel-Morgenstern dependence from the Gumbel-Barnett dependence, for different Kendall’s *τ * (*n=20,000*)

Kendall’s *τ *	$\vartheta _0^{FGM}$	$\vartheta _0^{GB}$	0.1	0.05	0.01
0	0	0	8	4	0
−0.05	−0.225	0.105	6	4	0
−0.1	−0.45	0.220	9	3	0
−0.15	−0.675	0.347	14	7	0
−0.2	−0.9	0.482	28	16	3
-$0.\bar{2}$	-1	0.547	35	18	5

Consider the FGM copula again as part of $H_0$. We simulate now with Algorithm 5, but let in the population $X_i$ marginally follow a Weibull distribution where the first parameter is again *θ * and the second parameter is *k* and models the deviation from the exponential distribution (where *k=1* constitutes the exponential). As Fig. 3 (right) shows, the test has quickly power where *k* deviates from 1. For Tables 3 and 5 we can report runtimes ranging between three and four hours for any of the *ϑ *.

## Example: enterprise lifespans in Germany

We aim at testing whether enterprise lifespans in Germany can be described by an exponential distribution. We are aware of the possible mis-specification with respect to the chosen marginal distribution of *T* and with respect to two copulas which we will allow for.

### Population, measurement and observability

The population comprises enterprises based in Germany and founded between January 1, 1990, and December 31, 2013 (*G = 24*). The age of the enterprise *i* at the end of the foundation period $T_i$ is in [0, 24], the support of the uniform distribution. The variable of interest, $X_i>0$, is the age of enterprise *i* at its closure. In the three years, from January 1, 2014, to December 31, 2016 (*s = 3*) enterprise closures are observed, together with their foundation dates, yielding a doubly truncated sample of $m_n = 55{,}279$ units. Even though further causes of enterprise closure exit, we consider insolvency as sole reason. Insolvency is a precise measurement in type and time. Enterprises file for insolvency at court, on the one hand, in order to receive financial aid for their employees and, on the other hand, can be punished for delayed filing of insolvency. Considered as competing risk model, the life expectancy of enterprises will not change due to further causes if the intensity of closure due to insolvency is equal to the intensity due to any other reason. Studies with other definitions of closure have shown that the definition has only limited impact on the parameter estimation (Sieg et al. 2025; Scholz and Weißbach 2026).

### Results for independence and dependence

The two resulting tests for the models of Sects. 5.1 and 5.2 are now displayed in their two components, the test statistic and the critical value. We compute the two test statistics with Algorithm 1. For the observations (55,279), the projections onto the upper (15,268) and right edge (6,768), as well as the intersection points (5,971,513), are determined. A total of 6,048,849 evaluations are therefore required. For $F^{\text {obs}}_{\hat{\boldsymbol{\theta }}_n}$ and $\alpha _{\hat{\boldsymbol{\theta }}_n}$, the parameter estimate is needed. Assuming independence, it is $\hat{\theta }_n^{\text {ind}}=0.08261$ (see Weißbach and Wied, 2022). (As a consequence, the observation probability is $\alpha _{\hat{\theta }_n}^{\text {ind}}=0.0955$.) Parameter estimation for FGM-dependence is $\hat{\theta }^{\text {FGM}}_n=0.08172$ and $\hat{\vartheta }^{\text {FGM}}_n=0.10256$ (see Toparkus and Weißbach , 2025). (The observation probability is $\alpha _{\hat{\boldsymbol{\theta }}_n}^{\text {FGM}}=0.09753$.) Intermediate values of step 7 in Algorithm 1 are now given in Table 6. In order to make the computational effort explicit, the real time duration of Algorithm 1 for either of the two models is of interest. Of course the burden scales with the hard- and software magnitude. The implementation of the test was carried out in R version 4.5.1 using RStudio. All computations were performed on a computer equipped with an Intel Core i7-7700HQ processor (2.80 GHz), 16 GB of RAM, and a graphics card with 4 GB of memory. The runtime for the model with dependence had been approximately three hours. Note that in R(Studio) (i) large amount of data can be stored more efficiently in data.table than in data.frame and (ii) that multiple loops can be avoided by vectorized operations.

**Table 6:** Auxiliary results for KS statistic according to Algorithm 1 for independence $C^{\text {ind}}$ and FGM copula $C^{\text {FGM}}_{\vartheta }$, $\times 10^2$

	$\delta ^1$	$\delta ^2$	$\delta ^3$	$\delta ^4$	$\delta ^5$
$C^{\text {ind}}$	2.2725	2.2733	$3.6173 \cdot 10^{-3}$	$1.7721 \cdot 10^{-3}$	$1.7728 \cdot 10^{-3}$
$C^{\text {FGM}}_{\vartheta }$	2.3040	2.3039	$3.6173 \cdot 10^{-3}$	$1.7697 \cdot 10^{-3}$	$1.7702 \cdot 10^{-3}$

The large values $\delta ^1$ and $\delta ^2$ indicate that the largest differences between empirical and hypothetical CDF occur in the observations or in the the intersection points. By multiplying the maximum by $\sqrt{m_n}$ and $\sqrt{\alpha _{\hat{\theta }_n}^{\text {ind}}}$ or $\sqrt{\alpha _{\hat{\boldsymbol{\theta }}_n}^{\text {FGM}}}$, the resulting test statistics are given in Table 7.

We now calculate the two critical values with Algorithm 2. Both intervals are divided in 100 segments, so that the grid has $10^4$ points and the number of entries of the covariance matrix becomes $10^4 \cdot 10^4 = 10^8$. The procedure is repeated 1,000 times, and for instance at level 99%, quantiles of 0.3558 for independence, and of 0.2914 for FGM-dependence, result. Table 7 contains quantiles at levels 90%, 95% and 99%.

**Table 7:** Critical values according to Algorithm 2 (three nominal levels) for independence (with the covariances (15)) and for $\text {FGM}$ copula (with the covariances (17))

Estimators Level *α *	0.10	0.05	0.01	0.10	0.05	0.01
	$C^{\text {ind}}$: (6) = 1.65			$C^{\text {FGM}}_{\vartheta }$: (6) = 1.69		
$(\hat{\theta }_n,n)$	0.5719	0.6558	0.8298	0.7320	0.8510	1.0816
$(\hat{\theta }_n,\hat{n})$	0.2869	0.3051	0.3558	0.2378	0.2535	0.2914

The test rejects clearly at any of those levels. The rejected independence of Toparkus and Weißbach (2025), suggest that the first test is of little use. For the second test, note again that rejection could also result for a non-uniform distribution of the foundation date. However, Dörre (2020, Lemma 2) shows that the assumption of a homogeneous Poisson process as a model for foundation (with whatever parameter) results in a uniform distribution of *T*, so that the exponential distribution assumption for *X* is most likely the (or one) reason for rejection. (Table 7 also lists quantiles for $(\hat{\boldsymbol{\theta }}_n, n)$ where it is itself interesting that the quantiles can be given in abundance of the estimation of *n*. Of course, the test statistics remain the same, but the quantiles more than double, so that the knowledge of *n* appears to do more harm than good.)

## Conclusion

Before we conclude the text with some limitations of our analysis, we like to stress one positive by-product. The asymptotic approximation of the test statistic with a Gaussian process in Theorem 1 is not only valid for the supremum as measures of discrepancy, but also for other global measures of discrepancy, for instance $\int \mathbb {G}_n(g)^2\,\textrm{d}\mathbb {P}_{\boldsymbol{\theta }_0}$ leads to the Cramér-von-Mises statistic.

On the other hand, the capacity of our statistical analysis to be transferred to other questions and retrospective data sets, can be limited by the involved approximations. To start with, the chosen bivariate population model is obvious only a rough approximation to the truth. (For instance, Weißbach and Dörre (2022) consider non-uniform foundation, but without respecting truncation dependence.) And another approximation is the sampling design, because our data are truly observational. The chosen truncation design had been proposed by Andersen et al. (1988), in order to respect the fact that the model of random variables for lifespan and birthdate in retrospective studies, implies a random number of observations. In short, the observations are considered as truncated simple random sample (denoted as “clockwise” design in Toparkus and Weißbach, 2025, Figure 2). To the contrary, Emura et al. (2017) and Dörre et al. (2021) view the observations as a sample of a truncated population (the “anti-clockwise” design). Without much evidence being given here, we found that, at least in the special case of left-truncation and known *n*, our goodness-of-fit test in the clockwise sense, behaves similarly to the goodness-of-fit test in the anti-clockwise design of Emura and Konno (2012).

When relaxing limitations, one must note that function $\phi _{\boldsymbol{\theta }_0}$ results from both the population model and the sampling design. So that any generalization must respect the first requirement in Assumption (A5), the finiteness of $\phi _{\boldsymbol{\theta }_0}$, as long as the theory should not be extended. (Note that this particular assumption is the only assumption in addition to the parametric analyses in Weißbach and Wied (2022) and Toparkus and Weißbach (2025).)

Some other potential approximation errors, especially with relation to the sample size, have been discussed in Sect. 6 in more detail, however still without being comprehensive. For instance, the approximation of the data as truncated point process with a Poisson process has not been assessed so far. It is theoretically known only to be good for small sample sizes, relative to the population size. (The ten percent of our application appears to be acceptable.) And to close with potential approximation errors related to the computational run time, we remind that the derivation of the covariance function for dependent truncation involves numerical integration. And as further benefit from numerical mathematics, Algorithm 2 uses the Cholesky decomposition and its cubic complexity is relatively slow, whereas Liu et al. (2019) present an overview of several other methods with lower complexity, partly at the expense of accuracy.

## Data Availability

The datasets analysed during the current study are available from the corresponding author on reasonable request.

## Appendix A: Proof of Corollary 2, Lemmas 1 and 2

### Proof of Corollary 2

By definition $\mathbb {E}_{\boldsymbol{\theta }_0}(g_{x,t,D})=\int _{[0,x]\times [0,t]\cap D} f_{\boldsymbol{\theta }_0}(\tilde{x}, \tilde{t}) \,\textrm{d}(\tilde{x}, \tilde{t})$ it is

$$\begin{aligned}&\lim _{\Vert {\textbf {h}} \Vert \searrow 0} \left\| \frac{\mathbb {E}_{\boldsymbol{\theta }_0 + {\textbf {h}}} - \mathbb {E}_{\boldsymbol{\theta }_0}}{\Vert {\textbf {h}}\Vert } - \frac{{\textbf {h}}^{\text {T}}\mathbb {E}_{\boldsymbol{\theta }_0}'}{\Vert {\textbf {h}}\Vert } \right\| _{\mathcal {G}_D} \\&= \lim _{\Vert {\textbf {h}} \Vert \searrow 0} \sup _{(x,t) \in S} \left| \int _{[0,x]\times [0,t]\cap D} \frac{f_{\boldsymbol{\theta }_0+{\textbf {h}}}(\tilde{x}, \tilde{t})-f_{\boldsymbol{\theta }_0}(\tilde{x}, \tilde{t})- {\textbf {h}}^{\text {T}}\dot{f}_{\boldsymbol{\theta }_0}(\tilde{x},\tilde{t})}{\Vert h\Vert } \,\textrm{d}(\tilde{x}, \tilde{t})\right| \\&\le \lim _{\Vert {\textbf {h}} \Vert \searrow 0} \int _{D} \left| \frac{f_{\boldsymbol{\theta }_0+h}(\tilde{x}, \tilde{t})-f_{\boldsymbol{\theta }_0}(\tilde{x}, \tilde{t})- {\textbf {h}}^{\text {T}}\dot{f}_{\boldsymbol{\theta }_0}(\tilde{x},\tilde{t})}{\Vert {\textbf {h}} \Vert } \right| \,\textrm{d}(\tilde{x}, \tilde{t})=0. \end{aligned}$$

The last equation results from interchanging *łim * and *∫ * which is possible by Scheffé’s lemma (and results about CDFs converging when densities do) and finally by applying the differentiability of $f_{\boldsymbol{\theta }}$ at $\boldsymbol{\theta }_0$ (by Assumption (A2)). Consequently, $\mathbb {E}_{\boldsymbol{\theta }_0}'$ is the Fréchet derivative of $\boldsymbol{\theta }\in [\varepsilon _{\theta }, 1/\varepsilon _{\theta }] \mapsto \mathbb {E}_{\boldsymbol{\theta }} \in \ell ^{\infty }(\mathcal {G}_D)$ at $\boldsymbol{\theta }_0$, because it is bounded by the continuity of $\dot{f}_{\boldsymbol{\theta }_0}$ due to Assumption (A2) and compactness of *D*.

### Proof of Lemma 1

First, note that according to Bauer (2001, Corollary 16.3), it follows from (A2) that $\boldsymbol{\theta }\mapsto \alpha _{\boldsymbol{\theta }}$ is continuously differentiable at $\boldsymbol{\theta }_0$. Because of $\alpha _{\boldsymbol{\theta }} \in (0,1)$ from (A1), the map $\boldsymbol{\theta }\mapsto \sqrt{\alpha _{\boldsymbol{\theta }}}$ is continuously differentiable at $\boldsymbol{\theta }_0$ too. Hence, it is

$$\begin{aligned} \sqrt{\alpha _{\boldsymbol{\theta }}}-\sqrt{\alpha _{\boldsymbol{\theta }_0}}-(\boldsymbol{\theta }-\boldsymbol{\theta }_0)^{\text {T}} \cdot \frac{1}{2\sqrt{\alpha _{\boldsymbol{\theta }_0}}} \dot{\alpha }_{\boldsymbol{\theta }_0}= o(\Vert \boldsymbol{\theta }-\boldsymbol{\theta }_0\Vert ). \end{aligned}$$

With the consistency of Assumption (A3) and van der Vaart (1998, Lemma 2.12) the equation

$$\begin{aligned} \sqrt{\alpha _{\hat{\boldsymbol{\theta }}_n}}-\sqrt{\alpha _{\boldsymbol{\theta }_0}}=(\hat{\boldsymbol{\theta }}_n-\boldsymbol{\theta }_0)^{\text {T}} \cdot \frac{\dot{\alpha }_{\boldsymbol{\theta }_0}}{2\sqrt{\alpha _{\boldsymbol{\theta }_0}}} + o_{\mathbb {P}_{\boldsymbol{\theta }_0}}(\Vert \hat{\boldsymbol{\theta }}_n -\boldsymbol{\theta }_0\Vert ) \end{aligned}$$

becomes evident also. The representation (11) follows from the asymptotic linearity (A4) with (A5) of the sequence $\sqrt{n}(\hat{\boldsymbol{\theta }}_n-\boldsymbol{\theta }_0)$. Thereby, note that $o_{\mathbb {P}_{\boldsymbol{\theta }_0}}(1)+o_{\mathbb {P}_{\boldsymbol{\theta }_0}}(\Vert \hat{\boldsymbol{\theta }}_n -\boldsymbol{\theta }_0\Vert )=o_{\mathbb {P}_{\boldsymbol{\theta }_0}}(1)$.

### Proof of Lemma 2

The convergence can be shown in two steps:

A.1 $$\begin{aligned} \left\| \frac{\sqrt{n}}{\sqrt{M_n}} \mathbb {P}_n(g_{x,t,D})- \frac{\mathbb {E}_{\boldsymbol{\theta }_0}(g_{x,t,D})}{\sqrt{\alpha _{\boldsymbol{\theta }_0}}} \right\| _{\mathcal {G}_D} + \left\| \frac{\mathbb {E}_{\hat{\boldsymbol{\theta }}_n}(g_{x,t,D})}{\sqrt{\alpha _{\hat{\boldsymbol{\theta }}_n}}} - \frac{\mathbb {E}_{\boldsymbol{\theta }_0}(g_{x,t,D})}{\sqrt{\alpha _{\boldsymbol{\theta }_0}}} \right\| _{\mathcal {G}_D} \end{aligned}$$

Adding $\pm 1/\sqrt{\alpha _{\boldsymbol{\theta }_0}}$ in the first norm results in

$$\begin{aligned}&\left\| \left( \frac{\sqrt{n}}{\sqrt{M_n}}\pm \frac{1}{\sqrt{\alpha _{\boldsymbol{\theta }_0}}}\right) \mathbb {P}_n(g_{x,t,D})- \frac{\mathbb {E}_{\boldsymbol{\theta }_0}(g_{x,t,D})}{\sqrt{\alpha _{\boldsymbol{\theta }_0}}} \right\| _{\mathcal {G}_D}\\&\le \left| \frac{\sqrt{n}}{\sqrt{M_n}}- \frac{1}{\sqrt{\alpha _{\boldsymbol{\theta }_0}}}\right| \cdot \left\| \mathbb {P}_n(g_{x,t,D}) \right\| _{\mathcal {G}_D} + \frac{1}{\sqrt{\alpha _{\boldsymbol{\theta }_0}}} \left\| \mathbb {P}_n(g_{x,t,D})- \mathbb {E}_{\boldsymbol{\theta }_0}(g_{x,t,D}) \right\| _{\mathcal {G}_D}. \end{aligned}$$

From the LLN, we get $\vert \sqrt{n}/\sqrt{M_n}- \sqrt{\alpha _{\boldsymbol{\theta }_0}}^{-1}\vert \overset{P}{\longrightarrow }0$ and

$$\begin{aligned} \left\| \mathbb {P}_n(g_{x,t,D}) \right\| _{\mathcal {G}_D}=\sup _{(x,t) \in D} \vert \frac{1}{n}\sum _{i=1}^n 1\!\!1_{\{ [0,x]\times [0,t]\cap D\}} (X_i,T_i)^\text {T} \vert = M_n/n \overset{P}{\longrightarrow }\alpha _{\boldsymbol{\theta }_0}. \end{aligned}$$

As demonstrated in Corollary 1, the class $\mathcal {G}_D$ exhibits the characteristics of a Glivenko-Cantelli and a Donsker class. Hence, it can be deduced that

$$\begin{aligned} \left\| \mathbb {P}_n(g_{x,t,D})- \mathbb {E}_{\boldsymbol{\theta }_0}(g_{x,t,D}) \right\| _{\mathcal {G}_D} \overset{a.s.}{\longrightarrow }0. \end{aligned}$$

With respect to the second norm in (A.1), due to due to Corollary 2, the map $\boldsymbol{\theta }\in \Theta \mapsto \mathbb {E}_{\boldsymbol{\theta }} \in \ell ^{\infty }(\mathcal {G}_D)$ is Fréchet differentiable at $\boldsymbol{\theta }_0$ so that by definition

$$\begin{aligned} \Vert \mathbb {E}_{\boldsymbol{\theta }_0 + {\textbf {h}}}-\mathbb {E}_{\boldsymbol{\theta }_0} - {\textbf {h}}^{\text {T}} \dot{\mathbb {E}}_{\boldsymbol{\theta }_0} \Vert _{\mathcal {G}_D} = o(||{\textbf {h}} ||), \end{aligned}$$

with a bounded $\dot{\mathbb {E}}_{\boldsymbol{\theta }_0}$, so that $\Vert (\mathbb {E}_{\boldsymbol{\theta }_0 + {\textbf {h}}}-\mathbb {E}_{\boldsymbol{\theta }_0}) \Vert _{\mathcal {G}_D}/ ||{\textbf {h}} ||= O(1)$. And now

$$\begin{aligned} \Vert \mathbb {E}_{\boldsymbol{\theta }_0 + {\textbf {h}}}-\mathbb {E}_{\boldsymbol{\theta }_0}\Vert _{\mathcal {G}_D} = O(1) o(1)=o(1), \end{aligned}$$

i.e. continuity of $\mathbb {E}_{\boldsymbol{\theta }}$ at $\boldsymbol{\theta }_0$. Furthermore, the map $\boldsymbol{\theta }\mapsto 1/\sqrt{\alpha _{\boldsymbol{\theta }}}$ is continuous in $\boldsymbol{\theta }_0$ by Assumption (A1) and interpreted as a map to $\ell ^{\infty }(\mathcal {G}_D)$, it is constant. Hence, the composition $\boldsymbol{\theta }\mapsto \mathbb {E}_{\boldsymbol{\theta }}/\sqrt{\alpha _{\boldsymbol{\theta }}}$ is continuous in $\boldsymbol{\theta }_0$ also. The consistency $\hat{\boldsymbol{\theta }}_n \rightarrow \boldsymbol{\theta }_0$ in Assumption (A3) and continuous mapping theorem for metric spaces (van der Vaart, 1998, Theorem 18.11), together ensure that the second norm in (A.1) converges to zero in probability as $n \rightarrow \infty$.

## Appendix B: Proof of Theorem 1

The first step involves a series of approximations in terms of the norm $\ell ^{\infty }(\mathcal {G_D})$. For this purpose, the Lemma 1 allows a replacement in term $\textcircled {2}$ and the last summand in Lemma 2 replaces term $\textcircled {4}$ (both in (10)):

$$\begin{aligned} (8)=(10)=&\,\sqrt{n}\left( \mathbb {P}_n(g_{x,t,D})- \mathbb {E}_{\hat{\boldsymbol{\theta }}_n}(g_{x,t,D})\right) \\&+\left[ \frac{1}{\sqrt{n}} \sum _{i=1}^n \phi _{\boldsymbol{\theta }_0}(X_i,T_i)^{\text {T}}\cdot \frac{\dot{\alpha }_{\boldsymbol{\theta }_0}}{2\sqrt{\alpha _{\boldsymbol{\theta }_0}}}-\left( \sqrt{M_n}-\sqrt{n} \sqrt{\alpha _{\boldsymbol{\theta }_0}}\right) \right] \\&\cdot \left[ 2\, \mathbb {E}_{\boldsymbol{\theta }_0}(g_{x,t,D})/\sqrt{\alpha _{\boldsymbol{\theta }_0}}\right] +o_{\mathbb {P}_{\boldsymbol{\theta }_0}}(1) \end{aligned}$$

The linear representation of Lemma 1, as well as the observed sample size $M_n$ and the observation probability $\alpha _{\boldsymbol{\theta }_0}$, can be expressed in terms of the empirical process as well, namely

$$\begin{aligned}&\frac{1}{\sqrt{n}} \sum _{i=1}^n \phi _{\boldsymbol{\theta }_0}(X_i,T_i)=\sqrt{n}\big (\mathbb {P}_n(\phi _{\boldsymbol{\theta }_0})-\mathbb {E}_{\boldsymbol{\theta }_0}(\phi _{\boldsymbol{\theta }_0})\big ) \quad \text {and}\\&\sqrt{M_n}-\sqrt{n} \sqrt{\alpha _{\boldsymbol{\theta }_0}}=\sqrt{n}\left( \sqrt{\mathbb {P}_n(1\!\!1_D)} -\sqrt{\mathbb {E}_{\boldsymbol{\theta }_0}(1\!\!1_D)}\right) , \end{aligned}$$

where $\mathbb {E}_{\boldsymbol{\theta }_0}(\phi _{\boldsymbol{\theta }_0})=\boldsymbol{0}$ (as per (A5)). Inserting these two terms results in

B.1 $$\begin{aligned} \begin{aligned} (8)=&\,\sqrt{n}\left( \mathbb {P}_n(g_{x,t,D})- \mathbb {E}_{\hat{\boldsymbol{\theta }}_n}(g_{x,t,D})\right) \\&+\left[ \sqrt{n}\big (\mathbb {P}_n(\phi _{\boldsymbol{\theta }_0})-\mathbb {E}_{\boldsymbol{\theta }_0}(\phi _{\boldsymbol{\theta }_0})\big )^{\text {T}}\cdot \frac{\dot{\alpha }_{\boldsymbol{\theta }_0}}{2\sqrt{\alpha _{\boldsymbol{\theta }_0}}}-\sqrt{n}\left( \sqrt{\mathbb {P}_n(1\!\!1_D)} -\sqrt{\mathbb {E}_{\boldsymbol{\theta }_0}(1\!\!1_D)}\right) \right] \\&\cdot \left[ 2\, \mathbb {E}_{\boldsymbol{\theta }_0}(g_{x,t,D})/\sqrt{\alpha _{\boldsymbol{\theta }_0}}\right] +o_{\mathbb {P}_{\boldsymbol{\theta }_0}}(1). \end{aligned} \end{aligned}$$

The third and final approximation is assigned to term $\textcircled {1}$ of the expression (10). As previously mentioned, the convergence in distribution of this expression was proven in van der Vaart (1998, Theorem 19.23). The proof of this theorem is based on the following decomposition:

$$\begin{aligned} \sqrt{n}(&\mathbb {P}_n-\mathbb {E}_{\hat{\boldsymbol{\theta }}_n})(g_{x,t,D})=\sqrt{n}(\mathbb {P}_n-\mathbb {E}_{\boldsymbol{\theta }_0})(g_{x,t,D})-\sqrt{n}(\mathbb {E}_{\hat{\boldsymbol{\theta }}_n}-\mathbb {E}_{\boldsymbol{\theta }_0})(g_{x,t,D})\nonumber \\&=\sqrt{n}(\mathbb {P}_n-\mathbb {E}_{\boldsymbol{\theta }_0})(g_{x,t,D})- \sqrt{n}(\hat{\boldsymbol{\theta }}_n-\boldsymbol{\theta }_0)^{\text {T}}\cdot \dot{\mathbb {E}}_{\boldsymbol{\theta }_0}(g_{x,t,D})+o_{\mathbb {P}_{\boldsymbol{\theta }_0}}(1) \end{aligned}$$

Thereafter, the Fréchet differentiability of the function $\boldsymbol{\theta }\mapsto \mathbb {E}_{\boldsymbol{\theta }}$ from Corollary 2 and the asymptotic linearity of the estimation sequence $\sqrt{n}(\hat{\boldsymbol{\theta }}_n-\boldsymbol{\theta }_0)$ from Assumptions (A4) and (A5) are used. Rewriting the first summand in (B.1) leads to:

B.2 $$\begin{aligned} \begin{aligned}&\sqrt{n}\big (\mathbb {P}_n(g_{x,t,D})-\mathbb {E}_{\boldsymbol{\theta }_0}(g_{x,t,D})\big )-\sqrt{n}\big (\mathbb {P}_n(\phi _{\boldsymbol{\theta }_0})-\mathbb {E}_{\boldsymbol{\theta }_0}(\phi _{\boldsymbol{\theta }_0})\big )^{\text {T}} \cdot \dot{\mathbb {E}}_{\boldsymbol{\theta }_0}(g_{x,t,D})\\&+\Big [\sqrt{n}\big (\mathbb {P}_n(\phi _{\boldsymbol{\theta }_0})-\mathbb {E}_{\boldsymbol{\theta }_0}(\phi _{\boldsymbol{\theta }_0})\big )^{\text {T}} \cdot \frac{\dot{\alpha }_{\boldsymbol{\theta }_0}}{2\sqrt{\alpha _{\boldsymbol{\theta }_0}}}-\sqrt{n}\left( \sqrt{\mathbb {P}_n(1\!\!1_D)} -\sqrt{\mathbb {E}_{\boldsymbol{\theta }_0}(1\!\!1_D)}\right) \Big ]\\&\quad \cdot \left[ 2\, \mathbb {E}_{\boldsymbol{\theta }_0}(g_{x,t,D})/\sqrt{\alpha _{\boldsymbol{\theta }_0}}\right] +o_{\mathbb {P}_{\boldsymbol{\theta }_0}}(1) \end{aligned} \end{aligned}$$

The objective is now to derive the limiting distribution of the process delineated in (B.2). Corollary 1 states that $\mathcal {G}_D$ is a Donsker class and, with Assumption (A5), it is $\Vert \phi _{\boldsymbol{\theta }_0}\Vert _{\infty } < \infty$. By means of elementary considerations about the bracketing numbers, the finite class $\{\phi _{\boldsymbol{\theta }_0}\}$ is Donsker, too. As demonstrated in van der Vaart and Wellner (1996,  Example 2.10.7), the Donsker property of two classes extends to their union with $\mathcal {G}_D^{\phi }:=\mathcal {G}_D \cup \{\phi _{\boldsymbol{\theta }_0}\}$. Accordingly, the sequence $\{\sqrt{n}(\mathbb {P}_n-\mathbb {E}_{\boldsymbol{\theta }_0})(g_{x,t,D}): \, g_{x,t,D} \in \mathcal {G}_D^{\phi }\}$ converges in distribution in $\ell ^{\infty }(\mathcal {G}_D^{\phi })$ to a $\mathbb {P}_{\boldsymbol{\theta }_0}$-Brownian bridge $\mathbb {B}_{\mathbb {P}_{\boldsymbol{\theta }_0}}$. The map

$$\begin{aligned} \varphi : \left( \sqrt{n}(\mathbb {P}_n-\mathbb {E}_{\boldsymbol{\theta }_0})\right) \mapsto \begin{pmatrix} \sqrt{n}\big (\mathbb {P}_n-\mathbb {E}_{\boldsymbol{\theta }_0}\big ) \\ \sqrt{n}\big (\mathbb {P}_n(1\!\!1_D)-\mathbb {E}_{\boldsymbol{\theta }_0}(1\!\!1_D)\big )\\ \sqrt{n}\big (\mathbb {P}_n(\phi _{\boldsymbol{\theta }_0,1})-\mathbb {E}_{\boldsymbol{\theta }_0}(\phi _{\boldsymbol{\theta }_0,1})\big )\\ \sqrt{n}\big (\mathbb {P}_n(\phi _{\boldsymbol{\theta }_0,2})-\mathbb {E}_{\boldsymbol{\theta }_0}(\phi _{\boldsymbol{\theta }_0,2})\big ) \end{pmatrix} \end{aligned}$$

is continuous for all $(\sqrt{n}(\mathbb {P}_n-\mathbb {E}_{\boldsymbol{\theta }_0})) \in \ell ^{\infty }(\mathcal {G}_D^{\phi })$. Therefore, the continuous mapping theorem for metric spaces can be applied to establish the convergence in distribution of $\{\varphi \big (\sqrt{n}(\mathbb {P}_n-\mathbb {E}_{\boldsymbol{\theta }_0})\big )(g_{x,t,D}): \, g_{x,t,D} \in \mathcal {G}_D^{\phi }\}$ in $\ell ^{\infty }(\mathcal {G}_D^{\phi })$ to $\varphi (\mathbb {B}_{\mathbb {P}_{\boldsymbol{\theta }_0}})$, i.e. to ($n \rightarrow \infty$) $\big (\mathbb {B}_{\mathbb {P}_{\boldsymbol{\theta }_0}},\mathbb {B}_{\mathbb {P}_{\boldsymbol{\theta }_0}}(1\!\!1_D),\mathbb {B}_{\mathbb {P}_{\boldsymbol{\theta }_0}}(\phi _{\boldsymbol{\theta }_0,1}),\mathbb {B}_{\mathbb {P}_{\boldsymbol{\theta }_0}}(\phi _{\boldsymbol{\theta }_0,2})\big )^{\text {T}}$.

A comparison of the function *φ * with Eq. (B.2) reveals that the incorporation of the missing transformations by the root function necessitates the utilization of the functional delta method (van der Vaart, 1998, Theorem 20.8). Here, smoothness uses the Hadamard derivative, and a simplified result given in Appendix C. For this purpose, consider the function

$$\begin{aligned} \phi _{\Delta }:( \ell ^{\infty }(\mathcal {G}_D^{\phi }))^4 \rightarrow (\ell ^{\infty }(\mathcal {G}_D^{\phi }))^4, \quad \phi _{\Delta }(x,y,z_1,z_2)=(x,\sqrt{y},z_1,z_2)^{\text {T}}, \end{aligned}$$

which is Hadamard differentiable according to Lemma 4, correspondingly extended to four dimensions, with $\phi '_{\Delta }(x,y,z_1,z_2)=\text {diag}(1,(2\sqrt{y})^{-1},1,1)$. Given the convergence in distribution $\varphi (\sqrt{n}(\mathbb {P}_n-\mathbb {E}_{\boldsymbol{\theta }_0})) \rightarrow \varphi (\mathbb {B}_{\mathbb {P}_{\boldsymbol{\theta }_0}})$ in $\ell ^{\infty }(\mathcal {G}_D^{\phi })$ and the Hadamard differentiability of $\phi _{\Delta }$, we can employ the delta method. Using this method allows us to account for the transformation by the root function; however, our limiting distribution will be affected. It yields the sequence

$$\begin{aligned}&\sqrt{n}\left( \phi _{\Delta } \begin{pmatrix} \mathbb {P}_n \\ \mathbb {P}_n(1\!\!1_D)\\ \mathbb {P}_n(\phi _{\boldsymbol{\theta }_0,1})\\ \mathbb {P}_n(\phi _{\boldsymbol{\theta }_0,2}) \end{pmatrix} -\phi _{\Delta } \begin{pmatrix} \mathbb {E}_{\boldsymbol{\theta }_0} \\ \mathbb {E}_{\boldsymbol{\theta }_0}(1\!\!1_D)\\ \mathbb {E}_{\boldsymbol{\theta }_0}(\phi _{\boldsymbol{\theta }_0,1})\\ \mathbb {E}_{\boldsymbol{\theta }_0}(\phi _{\boldsymbol{\theta }_0,2}) \end{pmatrix} \right) \\&= \begin{pmatrix} \sqrt{n}\big (\mathbb {P}_n-\mathbb {E}_{\boldsymbol{\theta }_0}\big ) \\ \sqrt{n}\left( \sqrt{\mathbb {P}_n(1\!\!1_D)}-\sqrt{\mathbb {E}_{\boldsymbol{\theta }_0}(1\!\!1_D)}\,\right) \\ \sqrt{n}\big (\mathbb {P}_n(\phi _{\boldsymbol{\theta }_0,1})-\mathbb {E}_{\boldsymbol{\theta }_0}(\phi _{\boldsymbol{\theta }_0,1})\big )\\ \sqrt{n}\big (\mathbb {P}_n(\phi _{\boldsymbol{\theta }_0,2})-\mathbb {E}_{\boldsymbol{\theta }_0}(\phi _{\boldsymbol{\theta }_0,2})\big ) \end{pmatrix}\\&\rightarrow \phi _{\Delta }' \begin{pmatrix} \mathbb {E}_{\boldsymbol{\theta }_0} \\ \mathbb {E}_{\boldsymbol{\theta }_0}(1\!\!1_D)\\ \mathbb {E}_{\boldsymbol{\theta }_0}(\phi _{\boldsymbol{\theta }_0,1})\\ \mathbb {E}_{\boldsymbol{\theta }_0}(\phi _{\boldsymbol{\theta }_0,2}) \end{pmatrix} \cdot \begin{pmatrix} \mathbb {B}_{\mathbb {P}_{\boldsymbol{\theta }_0}} \\ \mathbb {B}_{\mathbb {P}_{\boldsymbol{\theta }_0}}(1\!\!1_D)\\ \mathbb {B}_{\mathbb {P}_{\boldsymbol{\theta }_0}}(\phi _{\boldsymbol{\theta }_0,1})\\ \mathbb {B}_{\mathbb {P}_{\boldsymbol{\theta }_0}}(\phi _{\boldsymbol{\theta }_0,2}) \end{pmatrix} = \begin{pmatrix} \mathbb {B}_{\mathbb {P}_{\boldsymbol{\theta }_0}} \\ \mathbb {B}_{\mathbb {P}_{\boldsymbol{\theta }_0}}(1\!\!1_D)/(2\sqrt{\alpha _{\boldsymbol{\theta }_0}})\\ \mathbb {B}_{\mathbb {P}_{\boldsymbol{\theta }_0}}(\phi _{\boldsymbol{\theta }_0,1})\\ \mathbb {B}_{\mathbb {P}_{\boldsymbol{\theta }_0}}(\phi _{\boldsymbol{\theta }_0,2}) \end{pmatrix}, \end{aligned}$$

which converges in distribution in $\ell ^{\infty }(\mathcal {G}_D^{\phi })^4$ as $n \rightarrow \infty$. Finally, the four components of the vector can be combined using again the continuous mapping theorem for metric spaces, which results in the expression given in (B.2). Under $H_0$, the sequence

$$\begin{aligned}&\Big \{\sqrt{n}\big (\mathbb {P}_n-\mathbb {E}_{\boldsymbol{\theta }_0}\big )-\sqrt{n}\big (\mathbb {P}_n(\phi _{\boldsymbol{\theta }_0})-\mathbb {E}_{\boldsymbol{\theta }_0}(\phi _{\boldsymbol{\theta }_0})\big )^{\text {T}} \cdot \dot{\mathbb {E}}_{\boldsymbol{\theta }_0}\\&+\Big [\sqrt{n}\big (\mathbb {P}_n(\phi _{\boldsymbol{\theta }_0})-\mathbb {E}_{\boldsymbol{\theta }_0}(\phi _{\boldsymbol{\theta }_0})\big )^{\text {T}} \cdot \frac{\dot{\alpha }_{\boldsymbol{\theta }_0}}{2\sqrt{\alpha _{\boldsymbol{\theta }_0}}}-\sqrt{n}\left( \sqrt{\mathbb {P}_n(1\!\!1_D)} -\sqrt{\mathbb {E}_{\boldsymbol{\theta }_0}(1\!\!1_D)}\right) \Big ]\\&\quad \cdot \left[ 2\, \mathbb {E}_{\boldsymbol{\theta }_0}/\sqrt{\alpha _{\boldsymbol{\theta }_0}}\right] (g_{x,t,D}): g_{x,t,D} \in \mathcal {G}_D^{\phi }\Big \} \end{aligned}$$

converges in distribution in $\ell ^{\infty }(\mathcal {G}_D^{\phi })$ and therefore also in $\ell ^{\infty }(\mathcal {G}_D)$ (see van der Vaart, 1998, Theorem 19.23) to the Gaussian process $\{\mathbb {H}_{\mathbb {P}_{\boldsymbol{\theta }_0}}(g_{x,t,D}):g_{x,t,D} \in \mathcal {G}_D \}$.

## Appendix C: Hadamard differentiability

Given that a function $\phi _{\Delta }$ is continuously differentiable in the common sense, the Hadamard differentiability for certain spaces is a direct consequence (see van der Vaart, 1998, Problem 20.6).

### Lemma 3

Let $\phi _{\Delta }:[0,1] \rightarrow \mathbb {R}$ be a continuously differentiable function with derivative $\phi _{\Delta }'$. Then, the map $z \mapsto \phi _{\Delta } \circ z$, whose domain is the set of functions $z:\mathcal {G} \rightarrow [0,1]$ contained in $\ell ^{\infty }(\mathcal {G})$, has for direction *h* the Hadamard derivative $\phi _{\Delta }'(z) h$.

### Lemma 4

Let $\phi ^1_{\Delta }:[0,1] \rightarrow \mathbb {R}$ be a continuously differentiable function. Further denote $\phi _{\Delta }(y_1,y_2)=(y_1, \phi ^1_{\Delta }(y_2))$ for $y_1, \,y_2 \in [0,1]$. Let the space $(\ell ^{\infty }(\mathcal {G})^2, \Vert \cdot \Vert _{\text {max}})$ be equipped with the maximum norm. Then, the mapping $z=(z_1,z_2) \mapsto \phi _{\Delta } \circ z$ for functions $z_1, \, z_2:\mathcal {G} \rightarrow [0,1]$ from $\ell ^{\infty }(\mathcal {G})$ is Hadamard differentiable with

$$\phi '_{\Delta }(z_1,z_2)=\begin{pmatrix} 1 & 0 \\ 0 & (\phi ^1_{\Delta })'(z_2) \end{pmatrix}.$$

### Proof

Fix $z=(z_1,z_2) \in \ell ^{\infty }(\mathcal {G})^2$. For *t → 0* and $h_t=(h_t^1,h_t^2) \rightarrow h=(h^1,h^2)$ it is

$$\begin{aligned}&\left\| \frac{\phi _{\Delta }(z+t h_t)-\phi _{\Delta }(z)}{t}-\phi _{\Delta }'(z)h\right\| _{\text {max}}\\&\quad =\left\| \begin{pmatrix} (z_1+th_t^1-z_1)/t \\ (\phi ^1_{\Delta }(z_2+th_t^2)-\phi ^1_{\Delta }(z_2))/t \end{pmatrix} - \begin{pmatrix} h^1\\ (\phi ^1_{\Delta })'(z_2)h^2 \end{pmatrix} \right\| _{\text {max}}\\&\quad =\max \left\{ \vert h_t^1-h^1\vert ,\left| (\phi ^1_{\Delta }(z_2+th_t^2)-\phi ^1_{\Delta }(z_2))/t-(\phi ^1_{\Delta })'(z_2)h^2\right| \right\} \rightarrow 0. \end{aligned}$$

For the last convergence, $\phi ^1_{\Delta }$ adopts the role of $\phi _{\Delta }$ in Lemma 3. *□ *

## Appendix D: Derivation of covariance function (13)

Write short $g_i:=g_{x_i,t_i,D}=1\!\!1_{[0,x_i]\times [0,t_i]\cap D} \in \mathcal {G}_D$ for *i=1, 2*. Recall Corollary 2 so that

$$\begin{aligned}&\begin{pmatrix} \dot{\mathbb {E}}_{\theta _0}(g_i)\\ \dot{\mathbb {E}}_{\vartheta _0}(g_i) \end{pmatrix} +\left[ \left( \mathbb {B}_{\mathbb {P}_{\boldsymbol{\theta }_0}}( \phi _{\boldsymbol{\theta }_0,1}), \mathbb {B}_{\mathbb {P}_{\boldsymbol{\theta }_0}}( \phi _{\boldsymbol{\theta }_0,2})\right) \cdot \begin{pmatrix} \dot{\alpha }_{\theta _0}\\ \dot{\alpha }_{\vartheta _0} \end{pmatrix} - \mathbb {B}_{\mathbb {P}_{\boldsymbol{\theta }_0}}(1\!\!1_D) \right] \cdot \frac{\mathbb {E}_{\boldsymbol{\theta }_0}(g_i)}{\alpha _{\boldsymbol{\theta }_0}}\\&=\mathbb {B}_{\mathbb {P}_{\boldsymbol{\theta }_0}} (g_i)+ \left( \mathbb {B}_{\mathbb {P}_{\boldsymbol{\theta }_0}}(\phi _{\boldsymbol{\theta }_0,1}),\mathbb {B}_{\mathbb {P}_{\boldsymbol{\theta }_0}}(\phi _{\boldsymbol{\theta }_0,2})\right) \cdot \left[ \frac{\mathbb {E}_{\boldsymbol{\theta }_0}(g_i)}{\alpha _{\boldsymbol{\theta }_0}} \begin{pmatrix} \dot{\alpha }_{\theta _0}\\ \dot{\alpha }_{\vartheta _0} \end{pmatrix} \right. \\&\left. - \begin{pmatrix} \dot{\mathbb {E}}_{\theta _0}(g_i)\\ \dot{\mathbb {E}}_{\vartheta _0}(g_i) \end{pmatrix}\right] -\mathbb {B}_{\mathbb {P}_{\boldsymbol{\theta }_0}}(1\!\!1_D) \cdot \frac{\mathbb {E}_{\boldsymbol{\theta }_0}(g_i)}{\alpha _{\boldsymbol{\theta }_0}}. \end{aligned}$$

To enhance readability, define further $K_{i,j}:=\left( \mathbb {E}_{\boldsymbol{\theta }_0}g_i\frac{\dot{\alpha }_{j}}{\alpha _{\boldsymbol{\theta }_0}}-\dot{\mathbb {E}}_{j}g_i\right)$, for *i=1,2* and $j \in \{\theta _0, \vartheta _0\}$. Thus, the covariance $\text {Cov}[\mathbb {H}_{\mathbb {P}_{\boldsymbol{\theta }_0}}g_1,\mathbb {H}_{\mathbb {P}_{\boldsymbol{\theta }_0}}g_2]$ is

$$\begin{aligned}&\text {Cov}\Bigg [\mathbb {B}_{\mathbb {P}_{\boldsymbol{\theta }_0}} g_1+\mathbb {B}_{\mathbb {P}_{\boldsymbol{\theta }_0}}\phi _{\boldsymbol{\theta }_0,1}\cdot K_{1,\theta _0}+\mathbb {B}_{\mathbb {P}_{\boldsymbol{\theta }_0}}\phi _{\boldsymbol{\theta }_0,2} \cdot K_{1,\vartheta _0}-\mathbb {B}_{\mathbb {P}_{\boldsymbol{\theta }_0}}1\!\!1_D\cdot \frac{\mathbb {E}_{\boldsymbol{\theta }_0}g_1}{\alpha _{\boldsymbol{\theta }_0}},\\&\hspace{28mm}\mathbb {B}_{\mathbb {P}_{\boldsymbol{\theta }_0}} g_2+\mathbb {B}_{\mathbb {P}_{\boldsymbol{\theta }_0}}\phi _{\boldsymbol{\theta }_0,1} \cdot K_{2, \theta _0}+\mathbb {B}_{\mathbb {P}_{\boldsymbol{\theta }_0}}\phi _{\boldsymbol{\theta }_0,2}\cdot K_{2,\vartheta _0}\\&\quad -\mathbb {B}_{\mathbb {P}_{\boldsymbol{\theta }_0}}1\!\!1_D \cdot \frac{\mathbb {E}_{\boldsymbol{\theta }_0}g_2}{\alpha _{\boldsymbol{\theta }_0}}\Bigg ]. \end{aligned}$$

The calculation of the covariance is performed sequentially by fixing one summand at a time and combining it with all opposing summands.

First summand:

$$\begin{aligned}&\text {Cov}\left[ \mathbb {B}_{\mathbb {P}_{\boldsymbol{\theta }_0}} g_1, \mathbb {B}_{\mathbb {P}_{\boldsymbol{\theta }_0}} g_2\right] +K_{2,\theta _0} \cdot \text {Cov}\left[ \mathbb {B}_{\mathbb {P}_{\boldsymbol{\theta }_0}} g_1, \mathbb {B}_{\mathbb {P}_{\boldsymbol{\theta }_0}} \phi _{\boldsymbol{\theta }_0,1} \right] \\&\quad +K_{2,\vartheta _0} \cdot \text {Cov}\left[ \mathbb {B}_{\mathbb {P}_{\boldsymbol{\theta }_0}} g_1, \mathbb {B}_{\mathbb {P}_{\boldsymbol{\theta }_0}} \phi _{\boldsymbol{\theta }_0,2} \right] -\frac{\mathbb {E}_{\boldsymbol{\theta }_0}g_2}{\alpha _{\boldsymbol{\theta }_0}}\cdot \text {Cov}\left[ \mathbb {B}_{\mathbb {P}_{\boldsymbol{\theta }_0}} g_1, \mathbb {B}_{\mathbb {P}_{\boldsymbol{\theta }_0}}1\!\!1_D \right] \end{aligned}$$

Second summand:

$$\begin{aligned}&K_{1,\theta _0}\cdot \text {Cov}\left[ \mathbb {B}_{\mathbb {P}_{\boldsymbol{\theta }_0}} g_2, \mathbb {B}_{\mathbb {P}_{\boldsymbol{\theta }_0}} \phi _{\boldsymbol{\theta }_0,1} \right] +K_{1,\theta _0}K_{2,\theta _0}\cdot \text {Cov}\left[ \mathbb {B}_{\mathbb {P}_{\boldsymbol{\theta }_0}} \phi _{\boldsymbol{\theta }_0,1}, \mathbb {B}_{\mathbb {P}_{\boldsymbol{\theta }_0}} \phi _{\boldsymbol{\theta }_0,1} \right] \\&\quad +K_{1,\theta _0}K_{2,\vartheta _0}\cdot \text {Cov}\left[ \mathbb {B}_{\mathbb {P}_{\boldsymbol{\theta }_0}} \phi _{\boldsymbol{\theta }_0,1}, \mathbb {B}_{\mathbb {P}_{\boldsymbol{\theta }_0}} \phi _{\boldsymbol{\theta }_0,2} \right] \\&-\frac{\mathbb {E}_{\boldsymbol{\theta }_0}g_2}{\alpha _{\boldsymbol{\theta }_0}}K_{1,\theta _0}\cdot \text {Cov}\left[ \mathbb {B}_{\mathbb {P}_{\boldsymbol{\theta }_0}} \phi _{\boldsymbol{\theta }_0,1}, \mathbb {B}_{\mathbb {P}_{\boldsymbol{\theta }_0}} 1\!\!1_D \right] \end{aligned}$$

Third summand:

$$\begin{aligned}&K_{1,\vartheta _0}\cdot \text {Cov}\left[ \mathbb {B}_{\mathbb {P}_{\boldsymbol{\theta }_0}} g_2, \mathbb {B}_{\mathbb {P}_{\boldsymbol{\theta }_0}} \phi _{\boldsymbol{\theta }_0,2} \right] +K_{1,\vartheta _0}K_{2,\theta _0}\cdot \text {Cov}\left[ \mathbb {B}_{\mathbb {P}_{\boldsymbol{\theta }_0}} \phi _{\boldsymbol{\theta }_0,1}, \mathbb {B}_{\mathbb {P}_{\boldsymbol{\theta }_0}} \phi _{\boldsymbol{\theta }_0,2} \right] \\&\quad +K_{1,\vartheta _0}K_{2,\vartheta _0}\cdot \text {Cov}\left[ \mathbb {B}_{\mathbb {P}_{\boldsymbol{\theta }_0}} \phi _{\boldsymbol{\theta }_0,2}, \mathbb {B}_{\mathbb {P}_{\boldsymbol{\theta }_0}} \phi _{\boldsymbol{\theta }_0,2} \right] \\&-\frac{\mathbb {E}_{\boldsymbol{\theta }_0}g_2}{\alpha _{\boldsymbol{\theta }_0}}K_{1,\vartheta _0}\cdot \text {Cov}\left[ \mathbb {B}_{\mathbb {P}_{\boldsymbol{\theta }_0}} \phi _{\boldsymbol{\theta }_0,2}, \mathbb {B}_{\mathbb {P}_{\boldsymbol{\theta }_0}} 1\!\!1_D \right] \end{aligned}$$

Fourth summand:

$$\begin{aligned}&-\frac{\mathbb {E}_{\boldsymbol{\theta }_0}g_1}{\alpha _{\boldsymbol{\theta }_0}}\cdot \text {Cov}\left[ \mathbb {B}_{\mathbb {P}_{\boldsymbol{\theta }_0}} g_2, \mathbb {B}_{\mathbb {P}_{\boldsymbol{\theta }_0}}1\!\!1_D \right] -\frac{\mathbb {E}_{\boldsymbol{\theta }_0}g_1}{\alpha _{\boldsymbol{\theta }_0}}K_{2,\theta _0}\cdot \text {Cov}\left[ \mathbb {B}_{\mathbb {P}_{\boldsymbol{\theta }_0}} \phi _{\boldsymbol{\theta }_0,1}, \mathbb {B}_{\mathbb {P}_{\boldsymbol{\theta }_0}}1\!\!1_D \right] \\&-\frac{\mathbb {E}_{\boldsymbol{\theta }_0}g_1}{\alpha _{\boldsymbol{\theta }_0}}K_{2,\vartheta _0}\cdot \text {Cov}\left[ \mathbb {B}_{\mathbb {P}_{\boldsymbol{\theta }_0}} \phi _{\boldsymbol{\theta }_0,2}, \mathbb {B}_{\mathbb {P}_{\boldsymbol{\theta }_0}}1\!\!1_D \right] \\&+\frac{\mathbb {E}_{\boldsymbol{\theta }_0}g_1\cdot \mathbb {E}_{\boldsymbol{\theta }_0}g_2}{\alpha _{\boldsymbol{\theta }_0}^2}\cdot \text {Cov}\left[ \mathbb {B}_{\mathbb {P}_{\boldsymbol{\theta }_0}}1\!\!1_D, \mathbb {B}_{\mathbb {P}_{\boldsymbol{\theta }_0}}1\!\!1_D \right] \end{aligned}$$

The individual covariances contained therein can be simplified as follows, using the covariance of the $\mathbb {P}_{\boldsymbol{\theta }_0}$-Brownian bridge from (7) with *i,j =1,2*:

$$\begin{aligned} \begin{array}{lll} \text {Cov}\left[ \mathbb {B}_{\mathbb {P}_{\boldsymbol{\theta }_0}} g_1, \mathbb {B}_{\mathbb {P}_{\boldsymbol{\theta }_0}} g_2\right] & =\mathbb {E}_{\boldsymbol{\theta }_0}g_1g_2-\mathbb {E}_{\boldsymbol{\theta }_0}g_1\mathbb {E}_{\boldsymbol{\theta }_0}g_2 & \\ \text {Cov}\left[ \mathbb {B}_{\mathbb {P}_{\boldsymbol{\theta }_0}} g_i, \mathbb {B}_{\mathbb {P}_{\boldsymbol{\theta }_0}} \phi _{\boldsymbol{\theta }_0,j}\right] & =\mathbb {E}_{\boldsymbol{\theta }_0}g_i\phi _{\boldsymbol{\theta }_0,j}-\mathbb {E}_{\boldsymbol{\theta }_0}g_i\mathbb {E}_{\boldsymbol{\theta }_0}\phi _{\boldsymbol{\theta }_0,j} & =\mathbb {E}_{\boldsymbol{\theta }_0}g_i\phi _{\boldsymbol{\theta }_0,j}\\ \text {Cov}\left[ \mathbb {B}_{\mathbb {P}_{\boldsymbol{\theta }_0}} g_i, \mathbb {B}_{\mathbb {P}_{\boldsymbol{\theta }_0}} 1\!\!1_D\right] & =\mathbb {E}_{\boldsymbol{\theta }_0}g_i 1\!\!1_D-\mathbb {E}_{\boldsymbol{\theta }_0}g_i\mathbb {E}_{\boldsymbol{\theta }_0} 1\!\!1_D & =(1-\alpha _{\boldsymbol{\theta }_0})\,\mathbb {E}_{\boldsymbol{\theta }_0}g_i\\ \text {Cov}\left[ \mathbb {B}_{\mathbb {P}_{\boldsymbol{\theta }_0}} \phi _{\boldsymbol{\theta }_0,i}, \mathbb {B}_{\mathbb {P}_{\boldsymbol{\theta }_0}} \phi _{\boldsymbol{\theta }_0,j}\right] & =\mathbb {E}_{\boldsymbol{\theta }_0}\phi _{\boldsymbol{\theta }_0,i}\phi _{\boldsymbol{\theta }_0,j}-\mathbb {E}_{\boldsymbol{\theta }_0}\phi _{\boldsymbol{\theta }_0,i}\mathbb {E}_{\boldsymbol{\theta }_0}\phi _{\boldsymbol{\theta }_0,j} & =\mathbb {E}_{\boldsymbol{\theta }_0}\phi _{\boldsymbol{\theta }_0,i}\phi _{\boldsymbol{\theta }_0,j}\\ \text {Cov}\left[ \mathbb {B}_{\mathbb {P}_{\boldsymbol{\theta }_0}} \phi _{\boldsymbol{\theta }_0,i}, \mathbb {B}_{\mathbb {P}_{\boldsymbol{\theta }_0}} 1\!\!1_D\right] & =\mathbb {E}_{\boldsymbol{\theta }_0}\phi _{\boldsymbol{\theta }_0,i}1\!\!1_D-\mathbb {E}_{\boldsymbol{\theta }_0}\phi _{\boldsymbol{\theta }_0,i}\mathbb {E}_{\boldsymbol{\theta }_0}1\!\!1_D & =\mathbb {E}_{\boldsymbol{\theta }_0}\phi _{\boldsymbol{\theta }_0,i}1\!\!1_D =0\\ \text {Cov}\left[ \mathbb {B}_{\mathbb {P}_{\boldsymbol{\theta }_0}} 1\!\!1_D, \mathbb {B}_{\mathbb {P}_{\boldsymbol{\theta }_0}} 1\!\!1_D\right] & =\mathbb {E}_{\boldsymbol{\theta }_0}1\!\!1_D^2-\mathbb {E}_{\boldsymbol{\theta }_0}1\!\!1_D\mathbb {E}_{\boldsymbol{\theta }_0}1\!\!1_D & =\alpha _{\boldsymbol{\theta }_0}(1-\alpha _{\boldsymbol{\theta }_0}) \end{array} \end{aligned}$$

The simplifications include $\mathbb {E}_{\boldsymbol{\theta }_0}\phi _{\boldsymbol{\theta }_0,i}1\!\!1_D =\mathbb {E}_{\boldsymbol{\theta }_0}\phi _{\boldsymbol{\theta }_0,i}=0$ (see Assumption (A4)) and $\mathbb {E}_{\boldsymbol{\theta }_0}1\!\!1_D=\alpha _{\boldsymbol{\theta }_0}$. Inserting the values of the covariances leads to:

First and fourth summand:

$$\begin{aligned}&\mathbb {E}_{\boldsymbol{\theta }_0}g_1g_2-\mathbb {E}_{\boldsymbol{\theta }_0}g_1\mathbb {E}_{\boldsymbol{\theta }_0}g_2+K_{2,\theta _0} \cdot \mathbb {E}_{\boldsymbol{\theta }_0}g_1\phi _{\boldsymbol{\theta }_0,1} +K_{2,\vartheta _0} \cdot \mathbb {E}_{\boldsymbol{\theta }_0}g_1\phi _{\boldsymbol{\theta }_0,2}\\&-\frac{\mathbb {E}_{\boldsymbol{\theta }_0}g_2}{\alpha _{\boldsymbol{\theta }_0}}\cdot (1-\alpha _{\boldsymbol{\theta }_0})\,\mathbb {E}_{\boldsymbol{\theta }_0}g_1 -\frac{\mathbb {E}_{\boldsymbol{\theta }_0}g_1}{\alpha _{\boldsymbol{\theta }_0}}\cdot (1-\alpha _{\boldsymbol{\theta }_0})\,\mathbb {E}_{\boldsymbol{\theta }_0}g_2 \\&+\frac{\mathbb {E}_{\boldsymbol{\theta }_0}g_1\cdot \mathbb {E}_{\boldsymbol{\theta }_0}g_2}{\alpha _{\boldsymbol{\theta }_0}^2}\cdot \alpha _{\boldsymbol{\theta }_0}(1-\alpha _{\boldsymbol{\theta }_0})\\&=\mathbb {E}_{\boldsymbol{\theta }_0}g_1g_2-\mathbb {E}_{\boldsymbol{\theta }_0}g_1\mathbb {E}_{\boldsymbol{\theta }_0}g_2/\alpha _{\boldsymbol{\theta }_0}+K_{2,\theta _0} \cdot \mathbb {E}_{\boldsymbol{\theta }_0}g_1\phi _{\boldsymbol{\theta }_0,1} +K_{2,\vartheta _0} \cdot \mathbb {E}_{\boldsymbol{\theta }_0}g_1\phi _{\boldsymbol{\theta }_0,2} \end{aligned}$$

Second and third summand:

$$\begin{aligned}&K_{1,\theta _0}\cdot \mathbb {E}_{\boldsymbol{\theta }_0}g_2\phi _{\boldsymbol{\theta }_0,1} +K_{1,\theta _0}K_{2,\theta _0}\cdot \mathbb {E}_{\boldsymbol{\theta }_0}\phi _{\boldsymbol{\theta }_0,1}\phi _{\boldsymbol{\theta }_0,1} +K_{1,\theta _0}K_{2,\vartheta _0}\cdot \mathbb {E}_{\boldsymbol{\theta }_0}\phi _{\boldsymbol{\theta }_0,1}\phi _{\boldsymbol{\theta }_0,2}\\&+K_{1,\vartheta _0}\cdot \mathbb {E}_{\boldsymbol{\theta }_0}g_2\phi _{\boldsymbol{\theta }_0,2} +K_{1,\vartheta _0}K_{2,\theta _0}\cdot \mathbb {E}_{\boldsymbol{\theta }_0}\phi _{\boldsymbol{\theta }_0,1}\phi _{\boldsymbol{\theta }_0,2} +K_{1,\vartheta _0}K_{2,\vartheta _0}\cdot \mathbb {E}_{\boldsymbol{\theta }_0}\phi _{\boldsymbol{\theta }_0,2}\phi _{\boldsymbol{\theta }_0,2} \end{aligned}$$

Recall $K_i=(K_{i,\theta _0}, K_{i,\vartheta _0})^{\text {T}}$, we finally obtain (13).

$$\begin{aligned} \text {Cov}[\mathbb {H}_{\mathbb {P}_{\boldsymbol{\theta }_0}}g_1,\mathbb {H}_{\mathbb {P}_{\boldsymbol{\theta }_0}}g_2]&= \mathbb {E}_{\boldsymbol{\theta }_0}g_1g_2-\mathbb {E}_{\boldsymbol{\theta }_0}g_1\mathbb {E}_{\boldsymbol{\theta }_0}g_2/\alpha _{\boldsymbol{\theta }_0}+K_2^{\text {T}} \mathbb {E}_{\boldsymbol{\theta }_0}[g_1\phi _{\boldsymbol{\theta }_0}]\\&\quad +K_1^{\text {T}}\mathbb {E}_{\boldsymbol{\theta }_0}[g_2 \phi _{\boldsymbol{\theta }_0}]+K_1^{\text {T}} \mathbb {E}_{\boldsymbol{\theta }_0}[\phi _{\boldsymbol{\theta }_0}\phi _{\boldsymbol{\theta }_0}^{\text {T}}]K_2. \end{aligned}$$

## Appendix E: Gradient of observation probability for FGM copula

For the first coordinate of $\dot{\alpha }_{\boldsymbol{\theta }}^{\text {FGM}}$ see that $\dot{\alpha }_{\theta }^{\text {FGM}}=\partial \alpha _{\boldsymbol{\theta }}^{\text {FGM}}/(\partial \theta ) = \text {d} \alpha _{\theta }^{\text {ind}}/(\text {d} \theta ) - G^{-1} \vartheta (\text {d} \beta _{\theta })/(\text {d} \theta ) + 2\,G^{-2} \vartheta (\text {d} \gamma _{\theta })/(\text {d} \theta )$ where the first summand is in Weißbach and Wied (2022, Corollary1) and:

$$\begin{aligned} \frac{\text {d} \beta _{\theta }}{\text {d} \theta }= & \frac{1}{G} \Bigg [ \frac{1}{\theta ^2}(e^{-\theta s} - 1)(e^{-\theta G} + 1) + \frac{1}{\theta } \left[ s e^{-\theta s}(e^{-\theta G} + 1) \right. \\ & \left. + (e^{-\theta s} - 1) G e^{-\theta G} \right] - \frac{1}{2\theta ^2}(e^{-2\theta s} - 1)(e^{-2\theta G} + 1) \\ & - \frac{1}{2\theta } \left[ 2s e^{-2\theta s}(e^{-2\theta G} + 1) + (e^{-2\theta s} - 1) 2G e^{-2\theta G} \right] \Bigg ] \\ \frac{\text {d} \gamma _{\theta }}{\text {d} \theta }= & \frac{2}{G^2} \Bigg [ - \frac{2}{\theta ^3}(1 - e^{-\theta s})(1 - e^{-\theta G}) + \frac{1}{2\theta ^3}(1 - e^{-2\theta s})(1 - e^{-2\theta G}) \\ & + \frac{1}{\theta ^2} \left[ s e^{-\theta s}(1 - e^{-\theta G}) + G e^{-\theta G}(1 - e^{-\theta s}) \right] \\ & - \frac{1}{4\theta ^2} \left[ 2s e^{-2\theta s}(1 - e^{-2\theta G}) + 2G e^{-2\theta G}(1 - e^{-2\theta s}) \right] \Bigg ] \end{aligned}$$

The second coordinate is $\dot{\alpha }_{\vartheta }^{\text {FGM}}=\partial \alpha _{\boldsymbol{\theta }}^{\text {FGM}}/(\partial \vartheta ) = - G^{-1} \beta _{\theta } + 2 G^{-2}\gamma _{\theta }$.

## Appendix F: Elements of covariance function for models in Sects. 5.1 and 5.2

Recall the general formula (13) of the covariance.

### For independence

With $$g_{x_i,t_i,D}=1\!\!1_{[0,x_i]\times [0,t_i]\cap D}$$, it is $$\mathbb {E}_{\theta _0}(g_{x_i,t_i,D})=)$$$$\mathbb {P}_{\theta _0}((X_1,T_1)^{\text {T}} \in [0,x_i]\times [0,t_i]\cap D)$$. We construct the intersection of the rectangle and the parallelogramm as a rectangle minus one or two triangles, depending on the position of $$(x_i,t_i)$$ relative to *D* (see Weißbach and Wied, 2022, Figure 1 (right)). With the following auxiliary functions, we calculate the expectation for every case, where $$func_1$$ is the integral of the density $$f_{\theta _0}$$ over the rectangle, and $$func_2$$ and $$func_3$$ are the integrals over the corresponding lower and upper triangles, respectively:

$$\begin{aligned}&func_1(x,t)=\frac{t}{G}(1-e^{-\theta _0x}), \quad func_3(t)=\frac{e^{-\theta _0 t}}{G \theta _0}-\frac{1-t \theta _0}{G \theta _0},\\&func_2(x)=\frac{e^{-\theta _0 x}}{G \theta _0}(s\theta _0-\theta _0x -1) +\frac{e^{-\theta _0 s}}{G \theta _0} \\&\mathbb {E}_{\theta _0}(g_{x_i,t_i,D})= \left\{ \begin{array}{l} func_1(x_i,t_i)-func_2(x_i) -func_3(t_i), (x_i,t_i) \in D, \, x_i>s\\ func_1(x_i,t_i)-func_3(t_i), (x_i,t_i) \in D, \, x_i \le s\\ func_1(t_i+s,t_i)-func_2(t_i+s)-func_3(t_i), t_i< x_i-s\\ func1(x_i,x_i)-func_2(x_i)-func_3(x_i), x_i > s, \, x_i< t_i\\ func_1(x_i,x_i)-func_3(x_i), x_i \le s, \, x_i < t_i \end{array}\right. \end{aligned}$$

The Fréchet derivative has the representation according to Corollary 2, with *x* and *t* replaced by $x_i$ and $t_i$. Therefore, we only have to derive our density with respect to *θ *, while the geometric considerations concerning the integration regions are as above:

$$\begin{aligned}&dfunc_1(x,t)=\frac{xt}{G}e^{-\theta _0x}, \quad dfunc_3(t)=\frac{1}{G \theta _0^2}\left( 1-e^{\theta _0 t} (1+\theta _0 t)\right) ,\\&dfunc_2(x)=\frac{e^{-\theta _0 x}}{G}\left( x^2-sx+\frac{x}{\theta _0} +{1}{\theta _0^2}\right) -\frac{e^{-\theta _0 s}}{G \theta _0}\left( s+\frac{1}{\theta _0}\right) \\&\dot{\mathbb {E}}_{\theta _0}(g_{x_i,t_i,D})= \left\{ \begin{array}{l} dfunc_1(x_i,t_i)-dfunc_2(x_i) -dfunc_3(t_i), (x_i,t_i) \in D, \, x_i>s\\ dfunc_1(x_i,t_i)-dfunc_3(t_i), (x_i,t_i) \in D, \, x_i \le s\\ dfunc_1(t_i+s,t_i)-dfunc_2(t_i+s)-dfunc_3(t_i), t_i< x_i-s\\ dfunc_1(x_i,x_i)-dfunc_2(x_i)-dfunc_3(x_i), x_i > s, \, x_i< t_i\\ dfunc_1(x_i,x_i)-dfunc_3(x_i), x_i \le s, \, x_i < t_i \end{array}\right. \end{aligned}$$

Note also that $\mathbb {E}_{\theta _0}(\phi _{\theta _0}^2) =-\mathbb {E}_{\theta _0}(\dot{\psi }_{\theta _0})^{-1}$ and $\mathbb {E}_{\theta _0}(g_{x_2,t_2,D} \phi _{\theta _0}) =K_i \mathbb {E}_{\theta _0}(\dot{\psi }_{\theta _0})^{-1}$, so that - inserting $K_i$ - (13) can be simplified to an expression that does only additionally contain $\mathbb {E}_{\theta _0}(\dot{\psi }_{\theta _0})$. According to the simple form of $\psi _{\theta _0}$ under independent truncation (14), we have:

$$\begin{aligned} \mathbb {E}_{\theta _0}(\dot{\psi }_{\theta _0})=\alpha _{\theta _0} \left( \frac{2}{\theta _0^2} -\frac{s^2 e^{-\theta _0s}}{(1-e^{-\theta _0 s})^2} - \frac{G^2 e^{-G \theta _0}}{(1-e^{G \theta _0})^2}\right) \end{aligned}$$

### For dependence ($\text {FGM}$-copula)

Adding the Farlie-Gumbel-Morgernstern dependence, the auxiliary functions depend on two parameters, *ϑ * and *θ *:

$$\begin{aligned}&func_1(x,t)=\frac{t}{G}\left( 1-e^{-\theta _0x}\right) \cdot \left[ 1 + \vartheta _0 e^{-\theta _0 x} \left( 1-\frac{t}{G}\right) \right] \\ func_2(x)&=\frac{s-x}{G}e^{-\theta _0 x}+\frac{1}{\theta _0 G} \left( e^{-\theta _0 s} - e^{-\theta _0 x}\right) \\&\quad -\frac{\vartheta _0}{G}(x-s) e^{-\theta _0 x}\cdot \left( 1-\frac{x-s}{G}\right) \cdot \left( e^{- \theta _0 x}-1\right) \\&\quad -\frac{\vartheta _0}{G}e^{-\theta _0 x}\left( 1-\frac{2 (x-s)}{G}\right) \cdot \left( \frac{1}{2} e^{- \theta _0 x} - 1\right) \\&\quad +\frac{\vartheta _0}{G} e^{-\theta _0 s} \left( \frac{1}{2} e^{- \theta _0 s} - 1\right) +\frac{2\vartheta _0}{G^2 \theta _0^2} e^{-\theta _0 x}\left( \frac{1}{4} e^{- \theta _0 x} - 1\right) \\&\quad -\frac{2\vartheta _0}{G^2 \theta _0^2}e^{-\theta _0 s} \left( \frac{1}{4} e^{- \theta _0 s} - 1\right) \\ func_3(t)&=\frac{t}{G} + \frac{1}{\theta _0 G} \left( e^{-\theta _0 t}-1\right) +\frac{\vartheta _0}{\theta _0 G} e^{-\theta _0 t}\cdot \left( 1-\frac{2t}{G} \right) \cdot \left( \frac{1}{2} e^{-\theta _0 t} -1 \right) \\&\quad + \frac{1}{2} \frac{\vartheta _0}{\theta _0 G}-\frac{2 \vartheta _0}{G^2 \theta _0^2} e^{-\theta _0 t} \left( \frac{1}{4} e^{-\theta _0 t} -1 \right) - \frac{3 \vartheta _0}{2G^2 \theta _0^2} \\&\mathbb {E}_{\boldsymbol{\theta }_0}(g_{x_i,t_i,D})= \left\{ \begin{array}{l} func_1(x_i,t_i)-func_2(x_i) -func_3(t_i), (x_i,t_i) \in D, \, x_i>s\\ func_1(x_i,t_i)-func_3(t_i), (x_i,t_i) \in D, \, x_i \le s\\ func_1(t_i+s,t_i)-func_2(t_i+s)-func_3(t_i), t_i< x_i-s\\ func_1(x_i,x_i)-func_2(x_i)-func_3(x_i), x_i > s, \, x_i< t_i\\ func_1(x_i,x_i)-func_3(x_i), x_i \le s, \, x_i < t_i \end{array}\right. \end{aligned}$$

Therefore, we have to include the derivations for both parameters, that is $\dot{\mathbb {E}}_{\vartheta _0}(g_{x_i,t_i,D})$ and $\dot{\mathbb {E}}_{\theta _0}(g_{x_i,t_i,D})$. The corresponding derivations for the auxiliary functions have been conducted with *R* and are omitted here. Due to the information matrix equality, we have $\mathbb {E}_{\boldsymbol{\theta }}(\dot{\psi }_{\boldsymbol{\theta }_0})^{-1}=-\mathbb {E}_{\boldsymbol{\theta }_0}(\psi _{\boldsymbol{\theta }_0}\psi _{\boldsymbol{\theta }_0}^{\text {T}})$ with $\psi _{\theta _0}$ from (16). The integrations therein have to be done numerically.
